# Polymer Vesicles with Integrated Photothermal Responsiveness

**DOI:** 10.1021/jacs.3c07134

**Published:** 2023-09-04

**Authors:** Yingtong Luo, Hanglong Wu, Xuan Zhou, Jianhong Wang, Süleyman Er, Yudong Li, Pascal L. W. Welzen, Roy A. J. F. Oerlemans, Loai K. E. A. Abdelmohsen, Jingxin Shao, Jan C. M. van Hest

**Affiliations:** † Bio-Organic Chemistry, Institute of Complex Molecular Systems (ICMS), Eindhoven University of Technology, P.O. Box 513, 5600 MB Eindhoven, The Netherlands; ‡ DIFFER - Dutch Institute for Fundamental Energy Research, De Zaale 20, 5612 AJ Eindhoven, The Netherlands

## Abstract

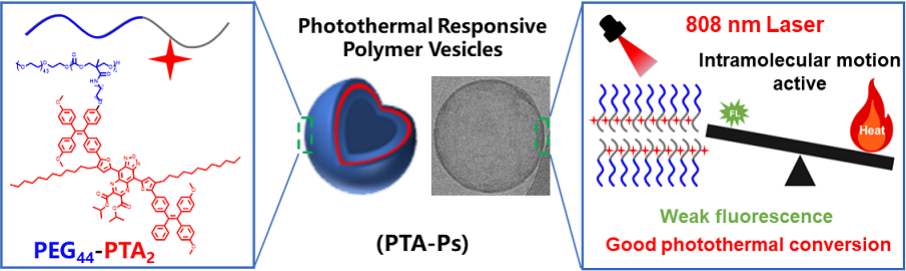

Functionalized polymer vesicles have been proven to be highly promising in biomedical applications due to their good biocompatibility, easy processability, and multifunctional responsive capacities. However, photothermal-responsive polymer vesicles triggered by near-infrared (NIR) light have not been widely reported until now. Herein, we propose a new strategy for designing NIR light-mediated photothermal polymer vesicles. A small molecule (PTA) with NIR-triggered photothermal features was synthesized by combining a D-D′-A-D′-D configuration framework with a molecular rotor function (TPE). The feasibility of the design strategy was demonstrated through density functional theory calculations. PTA moieties were introduced in the hydrophobic segment of a poly(ethylene glycol)-poly(trimethylene carbonate) block copolymer, of which the carbonate monomers were modified in the side chain with an active ester group. The amphiphilic block copolymers (PEG_44_-PTA_2_) were then used as building blocks for the self-assembly of photothermal-responsive polymer vesicles. The new class of functionalized polymer vesicles inherited the NIR-mediated high photothermal performance of the photothermal agent (PTA). After NIR laser irradiation for 10 min, the temperature of the PTA-Ps aqueous solution was raised to 56 °C. The photothermal properties and bilayer structure of PTA-Ps after laser irradiation were still intact, which demonstrated that they could be applied as a robust platform in photothermal therapy. Besides their photothermal performance, the loading capacity of PTA-Ps was investigated as well. Hydrophobic cargo (Cy7) and hydrophilic cargo (Sulfo-Cy5) were successfully encapsulated in the PTA-Ps. These properties make this new class of functionalized polymer vesicles an interesting platform for synergistic therapy in anticancer treatment.

## Introduction

Polymer vesicles are vesicular structures composed of a bilayer of amphiphilic block copolymers with much potential in nanomedicine due to their efficient cargo loading capacity and good biological stability.^1−4^ Recently, interest has been raised in the design and application of functionalized polymer vesicles with photosensitive and pH-responsive elements. With these additional responsive capacities, polymer vesicles can be more effectively employed in biomedical applications, such as cell/tissue imaging, photoactivated therapies, and pH-responsive drug release.^5−8^ In this regard, the application of polymer vesicles for photothermal therapy (PTT) has remained remarkably underexplored.^9−13^ PTT uses photosensitive molecules that upon irradiation lead to a local temperature rise, which, when performed in close proximity of target cells, induces apoptosis.^14−20^ PTT has an advantage compared to photodynamic therapy because no oxygen is required. Using polymer vesicles, not only the photothermal agents (PTAs) can be targeted to cells, but their loading capacity also allows the co-delivery of other therapeutic agents to develop synergistic therapies for anticancer therapy.^21,22^ To prevent premature release of the PTAs upon in vivo application, these units should preferably be integrated in the polymer vesicle architecture. Furthermore, to achieve deep penetration and weak absorption in biological tissue, near-infrared (NIR) light is an ideal candidate to be used as an external excitation light source when designing photothermal-responsive polymer vesicles (PTA-Ps).^23,24^

**Figure 1:**
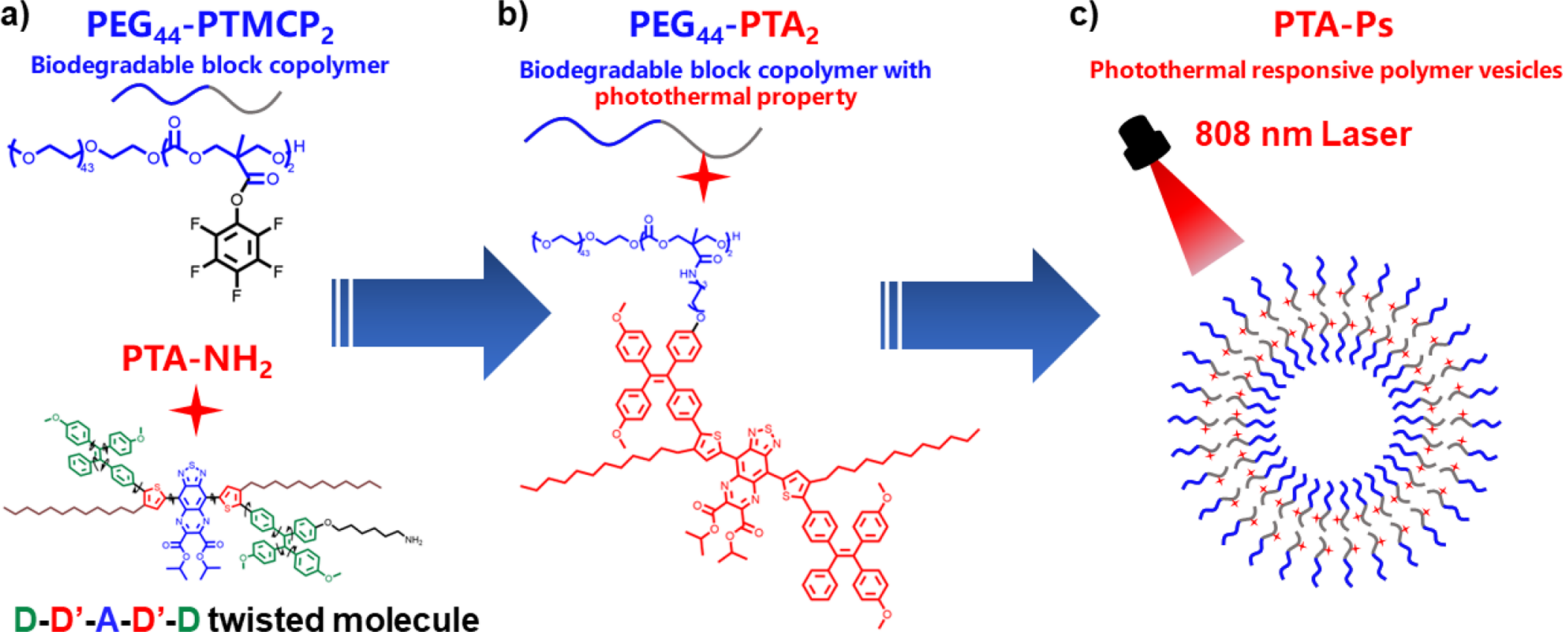
Synthetic scheme toward the preparation of photothermal polymer vesicles. (a) Structure of the amine-containing photothermal agent (PTA-NH_2_) and precursor copolymer (PEG_44_-PTMCP_2_). (b) Photothermal amphiphilic block copolymer (PEG_44_-PTA_2_). (c) Schematic depiction of PTA-Ps upon 808 nm laser irradiation.

In this work, a new type of amphiphilic block copolymer was designed and used for the construction of photothermal-responsive polymer vesicles. PTAs were synthesized according to a previously reported design strategy with a slight modification. The PTA was a D–D′–A–D′–D-type conjugated molecule with a twisted structure and effective photon absorption in the NIR window (Figure 1a).^25−28^ As amphiphilic block copolymer poly(ethylene glycol)-pol(trimethylene carbonate) was used, of which the carbonate monomers carried activated esters in the side chain. The PTAs were effectively conjugated, after which the polymers were self-assembled into polymer vesicles. Their photothermal features, stability, loading capacity with model drugs, and in vitro PTT were subsequently analyzed.

## Results and Discussion

To achieve a PTA with excellent photothermal properties and excitation potential in the NIR window, a careful design strategy was set up. As a basic structure for the PTA, an acrylate-substituted thiadiazoloquinoxaline (ATQ) was chosen as a strong electron-withdrawing acceptor, following conjugating to two thiophene moieties, which acted both as a strong donor and as a π-bridge to two second donor units.^29,30^ However, D–A–D-type conjugated structures usually are coplanar, which causes strong intermolecular interactions in the aggregated state, resulting in a significantly diminished heat generation capacity due to the quenching of the photoexcited state.^31^ Therefore, as secondary donors, alkoxy-substituted tetraphenylethylene (TPE) units were introduced with a twisted structure. Additionally, long alkyl chains were conjugated to the thiophenes as shielding units to further avoid strong intermolecular interactions (π–π interaction) and promote the intramolecular motion in the aggregated state.^27^ The design of this PTA was corroborated with density functional theory (DFT) calculations. Due to its biocompatibility, biodegradability, and chemical versatility, a modifiable poly(ethylene glycol)-poly(trimethylene carbonate) (PTMC) was selected as the amphiphilic polymer.^32,33^ It was demonstrated in previous research that PTMC-based amphiphilic block copolymers were effectively assembled into spherical polymer vesicles for a range of biomedical applications.^34−36^ To introduce the PTAs effectively, a functionalized PTMC copolymer with pentafluorophenyl ester side chains was envisaged (Figure 1a). Given that the PTA moieties contribute relatively strongly to the molecular weight of the block copolymer, it was decided to synthesize a precursor copolymer (PEG_44_-PTMCP_2_) with a relatively low degree of polymerization (DP) to achieve a balance between the hydrophilic and hydrophobic segments (Figure 1b). Thereafter, the as-prepared PEG_44_-PTA_2_ was self-assembled into polymer vesicles via the classic solvent exchange approach (Figure 1c).^35^

**Figure 2:**
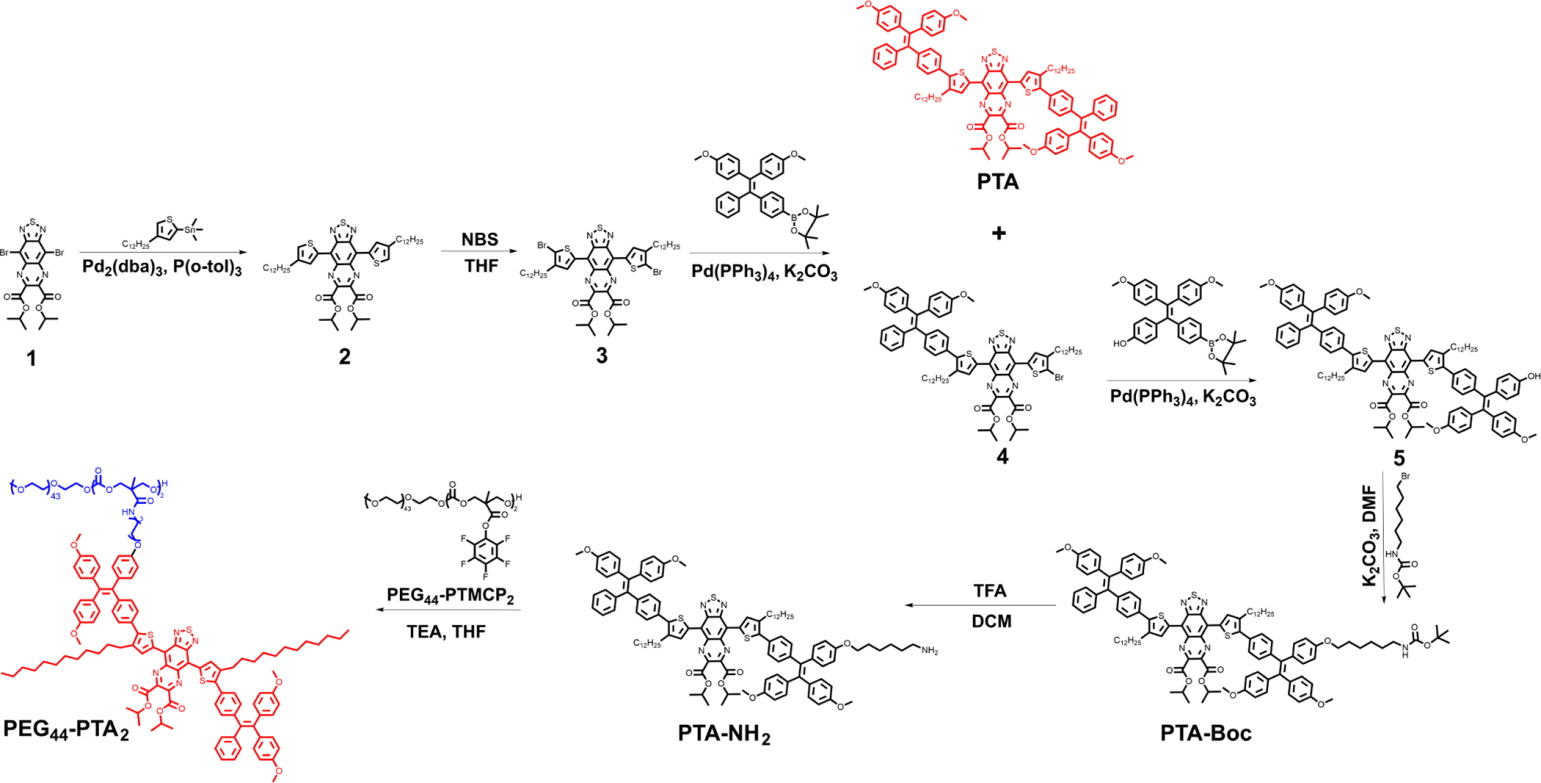
Synthetic route toward the PTA, its derivative (PTA-NH_2_), and photothermal amphiphilic block copolymer (PEG_44_-PTA_2_).

The overall synthetic routes of PTA and PEG_44_-PTA_2_ are shown in Figure 2. PTA and PTA-NH_2_ were synthesized from ATQ by a multiple-step procedure. First, 4-dodecyl-2-thiophene was introduced into the conjugated backbone by a Stille coupling reaction. Then, *N*-bromosuccinimide was used to provide the bromide reactive sites for conjugation with TPE. Finally, PTA was synthesized from compound **3** by a Suzuki coupling reaction. PTA-NH_2_ was synthesized according to the PTA synthesis route with some modifications. First, TPE-OH was introduced into compound **4** by a Suzuki coupling reaction. Then, 6-(Boc-amino)hexyl bromide was introduced into PTA via a nucleophilic substitution reaction. Finally, PTA-NH_2_ was obtained from PTA-Boc after deprotection. To prepare PEG_44_-PTA_2_, PEG_44_-PTMCP_2_ was synthesized by a ring-opening polymerization (ROP) of a biodegradable trimethylene carbonate derivative (TMCP), which was subsequently amidated with PTA-NH_2_.^35^ The overall yield of PEG_44_-PTA_2_ was 1.4%, and all synthesis and characterization details (^1^H NMR, ^13^C NMR, ^19^F NMR, MALDI-TOF-MS, and GPC analysis) can be found in the Supporting Information (Scheme S1, Figures S1–S22, and Table S1).

**Figure 3:**
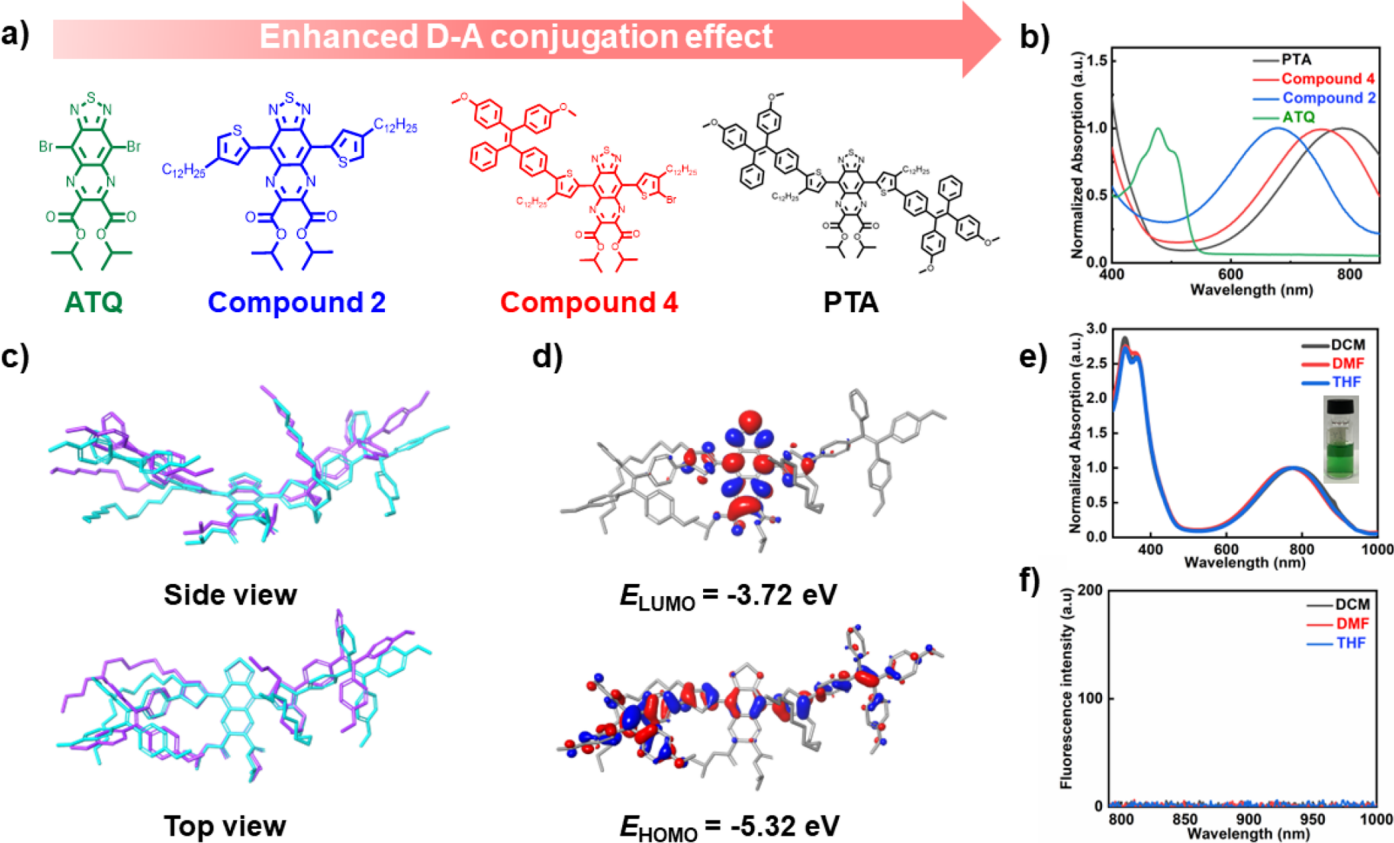
Structural and spectroscopic features of PTA and its precursor (ATQ, compound **2**, and compound **4**). (a) Molecular structure of ATQ, compound **2**, compound **4**, and PTA. (b) Absorption spectra of different conjugated systems, including ATQ, compound **2**, compound **4**, and PTA in DCM. (c) Overlap diagram of optimized ground state (S_0_) (blue) and excited state (S_1_) (purple) geometries of PTA. (d) Calculated frontier molecular orbitals of PTA. (e) Normalized UV–vis–NIR absorption spectra of PTA in different solvents (DCM, DMF, and THF). The inset is the optical image of PTA in DMF solution. (f) Emission spectra of PTA in different solvents (DCM, DMF, and THF, Ex = 780 nm).

To understand the relationship between the molecular structure and photophysical properties of PTA, DFT calculations were performed. First, the ground state (S_0_) and excited state (S_1_) geometric structures of PTA were optimized by DFT. Notably, the conjugated basic structure possessed a twisted shape with dihedral angles (27.8 and 34.7°) between the donor unit (thiophene) and the acceptor unit (ATQ) at S_0_ geometry. Furthermore, dihedral angles between the thiophene ring and the TPE unit were determined to be 51.4 and 46.4°, which allowed free motion of the TPE rotor, resulting in blocking of the radiative release channel and promoting photothermal conversion (Figure S23). It is noteworthy that the twist angles of α and α′ were significantly changed in the S_1_ state upon excitation, indicating that the molecular motion happened during the intramolecular charge transfer (ICT) process (Figure S23 and Figure 3c). Therefore, the highly twisted molecular skeleton not only limited intermolecular π–π interactions but also stimulated intramolecular motion, which is beneficial for improving the photothermal performance of PTA.^37−40^ Meanwhile, the long alkyl chains extending outward from the thiophene plane were employed as steric elements for the restriction of intermolecular π–π interactions.^27^ The highest occupied molecular orbital (HOMO) and lowest unoccupied molecular orbital (LUMO) mapping showed that the HOMO was delocalized along the conjugated backbone and the LUMO was centered on the electron-deficient ATQ core, revealing an apparent D–A interaction from the donor unit to the acceptor core (Figure 3d). In addition, as the ATQ core was strongly electron-deficient, PTA showed a narrow bandgap (1.60 eV). The narrow bandgap is beneficial for strong absorbance in the long-wavelength region.^38^ Furthermore, the intramolecular motion and strong ICT effect of PTA jointly facilitate the formation of a twisted intramolecular charge transfer (TICT) state, which can enhance the non-radiative decay. Altogether, these calculations confirmed the feasibility of the presented molecular design strategy. Subsequently, the photophysical properties of PTA and its precursor were studied. With the enhanced D–A effect of the conjugated backbone, the maximum absorption wavelength of PTA exhibited a significant bathochromic shift compared with its precursor (ATQ, compound **2**, and compound **4**) (Figure 3a,b). PTA dissolved in common organic solvents (DCM, DMF, and THF) exhibited a green color (Figure 3e, inset). Two absorption bands were observed, which were contributed to a high-energy π–π* transition and a low-energy ICT band. The maximum ICT absorption peak of PTA in DMF was at 785 nm, which was located at the NIR-I (ca. 700–900 nm) window.^23^ Moreover, the emission intensity of the conjugated molecule in common organic solvents was almost negligible, which indicated that the PTA with its strong TICT effect mainly releases energy through non-radiative pathways, namely, heat (Figure 3f). These theoretical and experimental results demonstrated the feasibility of our design strategy in the development of a photothermal molecule with light-harvesting ability in the NIR-I region.

**Figure 4:**
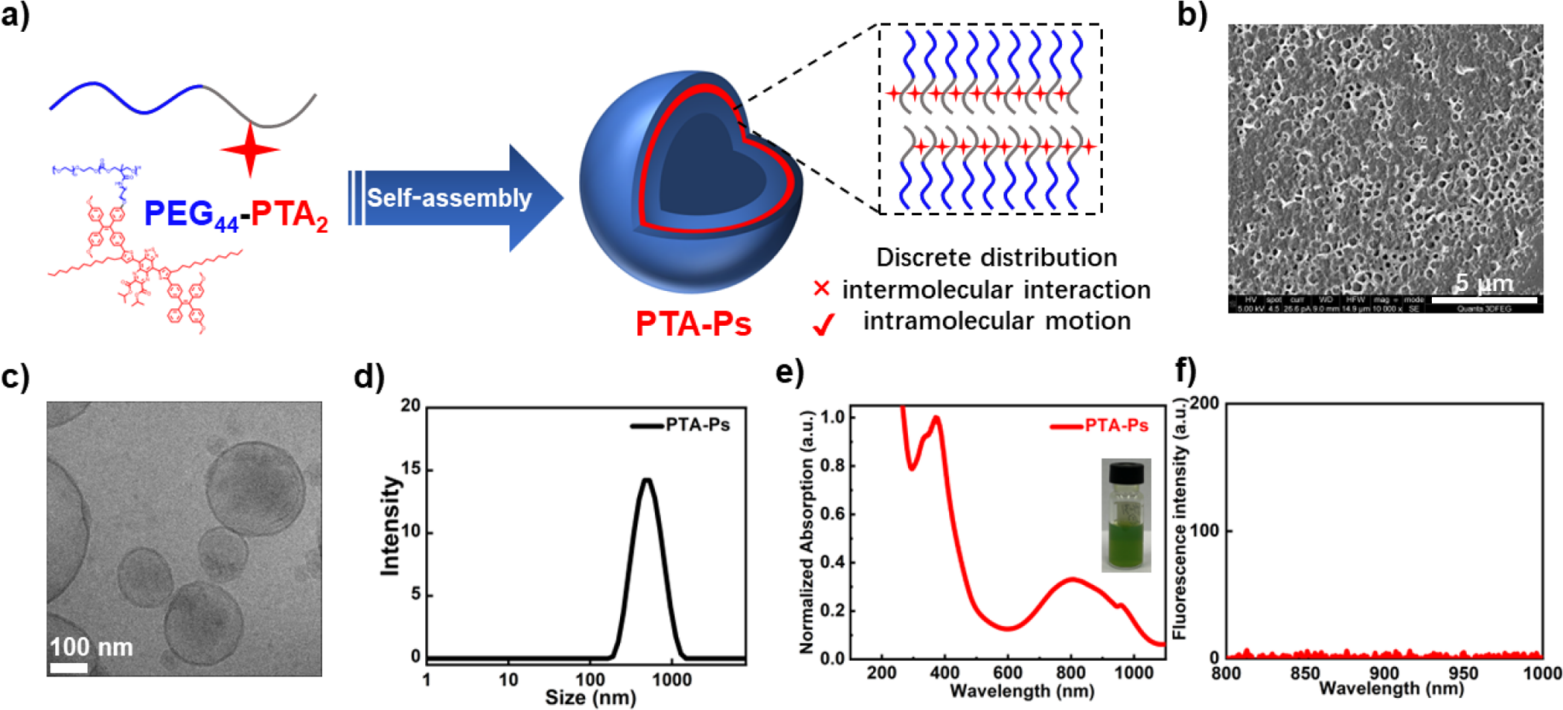
Preparation and characterization of PTA-Ps. (a) Schematic illustration of the preparation of PTA-Ps via self-assembly. (b) SEM images of PTA-Ps. Scale bar = 5 μm. (c) Cryo-TEM images of PTA-Ps. Scale bar = 100 nm. (d) Average size of PTA-Ps in water measured by DLS. (e) Normalized UV–vis–NIR absorption spectra of PTA-Ps in water. The inset is a picture of the aqueous solution of PTA-Ps. (f) Emission spectra of PTA-Ps in water (Ex = 780 nm).

To prepare photothermal-responsive polymer vesicles (PTA-Ps), the solvent switch method was used for self-assembly (Figure 4a). Briefly, the amphiphilic block copolymer was dissolved in THF, followed by addition of water at a fixed speed using a pump. Next, dialysis was conducted to remove the organic solvent. The as-prepared polymer vesicles were characterized using scanning electron microscopy (SEM), dynamic light scattering (DLS), and cryogenic transmission electron microscopy (Cryo-TEM). As shown in Figure 4b–d, a spherical morphology with a membrane thickness of ca. 6.1 ± 1.0 nm was obtained, and the average diameter was 443.1 ± 24.5 nm (PDI = 0.187 ± 0.045).

A vesicular morphology and evenly sized membrane structure suggest that the PTA moieties were distributed homogeneously in the bilayer of the polymer vesicles rather than accumulated together to form aggregates. The presence of the poly(trimethylene carbonate) backbone facilitated the isolation of the conjugated PTA units, which diminished intermolecular interactions and promoted the intramolecular motion in the assembled state.^41^

The successful formation of the vesicle structure also provided the space for cargo encapsulation. Within this hydrophobic membrane, the molecular dye Cy7 was effectively loaded as a hydrophobic cargo (Figure S24a). To prove the successful loading of Cy7, the emission wavelength of PTA-Ps before and after encapsulation with Cy7 dye was tested. The emission wavelength of cargo-loaded PTA-Ps was similar to the pure Cy7 solution, and the emission intensity of cargo-loaded PTA-Ps was significantly higher than that of unloaded polymer vesicles (Figure S24b,c). To evaluate the effect of hydrophobic cargo (Cy7) loading on the structure of the polymer vesicles, we have characterized the size and morphology of Cy7-loaded PTA-Ps using DLS and SEM. As shown in Figure S25a, the size of the PTA-Ps did not change significantly after loading with Cy7. The non-changed morphology of PTA-Ps after Cy7 encapsulation was observed with SEM (Figure S25b). Furthermore, Sulfo-Cy5 as a hydrophilic cargo was encapsulated in the aqueous core to further verify the loading capacity of PTA-Ps (Figure S26a). Sulfo-Cy5-loaded PTA-Ps exhibited a similar emission peak as pure Sulfo-Cy5 solution, whereas the emission intensity of pure PTA-Ps was almost negligible (Figure S26b,c). Moreover, PTA-Ps exhibited loading efficiencies of 2.9 and 3.8% and encapsulation efficiencies of 61.7 and 42.2% for Cy5 and Cy7, respectively (Figure S27). All these data confirmed the successful loading of the hydrophilic/hydrophobic cargo and demonstrated the possibility of using PTA-Ps as functional cargo carriers.

The optical features of the PTA moiety were maintained after its conjugation to the polymer (Figure S28). Upon self-assembly, the PTA-Ps still displayed a green color (Figure 4e, inset), although with a red-shifted absorption peak at 808 nm, which was conveniently very close to the commercial NIR-I laser (808 nm). Consequently, the 808 nm laser was selected as an external excitation light source for the subsequent photothermal performance tests. Moreover, the very low fluorescence intensity predicted that PTA-Ps would have excellent photothermal properties upon 808 nm laser irradiation (Figure 4f).

**Figure 5:**
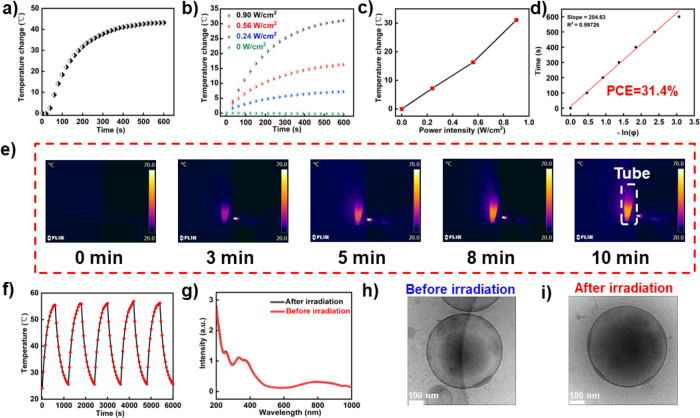
Photothermal properties of PTA and PTA-Ps. (a) Photothermal performance of PTA in DMF (1 mg/mL) upon NIR laser irradiation (808 nm, 0.90 W/cm^2^) for 10 min. (b) Temperature change of the PTA-Ps in water (1 mg/mL) under 808 nm laser irradiation for 10 min (0, 0.24, 0.56, and 0.90 W/cm^2^). (c) Temperature change of PTA-Ps in water (1 mg/mL) after 10 min irradiation with different power intensities (0, 0.24, 0.56, and 0.90 W/cm^2^). (d) Time constant for PTA-Ps heat transfer from the system, calculated with the linear time data from the system cooling period versus the negative natural logarithm of the system driving force temperature. The photothermal conversion efficiency (PCE) is 31.4%. (e) Corresponding infrared thermal mappings of PTA-Ps in water (1 mg/mL) upon 808 nm laser irradiation (0.90 W/cm^2^) as a function of time. (f) Photothermal stability of PTA-Ps in water (1 mg/mL) during five circles of heating–cooling. (g) UV–vis–NIR absorption spectra of PTA-Ps before and after 808 nm laser irradiation (0.90 W/cm^2^) for 10 min. (h, i) Cryo-TEM images of PTA-Ps before and after 808 nm laser irradiation (0.90 W/cm^2^, 10 min).

First, the temperature change upon NIR laser irradiation (808 nm) of a PTA solution in DMF (1 mg/mL) was investigated (Figure 5a). A temperature increase of 43 °C was achieved. The good photothermal performance was contributed to the non-radiative photothermal transition, as predicted by the DFT results.

To demonstrate that the photothermal features were maintained in the aggregated state, we tested the laser intensity-dependent temperature change of PTA-Ps upon NIR laser irradiation for 10 min. As shown in Figure 5b,c, the increased temperature was highly dependent on the incident laser power. With the increase of laser power, the maximum temperature of the PTA-Ps solution reached 56 °C (31 °C temperature increase). According to a previously published method, the photothermal conversion efficiency (PCE) of PTA-Ps was calculated, which was 31.4%. Additionally, to compare the photothermal performance of PTA-Ps with small molecule PTA, we also prepared PTA nanoparticles (PTA-NPs) via traditional nanoprecipitation methods.^29^ The DLS data proved that the average size of PTA-NPs is 72.2 ± 0.2 nm (PDI = 0.159 ± 0.013) (Figure S29). The PCE of PTA-Ps (31.4%) was higher than both these PTA-NPs (29.0%) and existing photothermal materials, such as gold nanorods (20.7%) (Figure 5d and Figures S30 and S31).^27^ The high PCE of PTA-Ps demonstrates that the free motion of the PTA was still retained in the aggregated state. Infrared thermal images further confirmed the heat generation ability of the PTA-Ps (Figure 5e). To exclude the influence from the photothermal effect of the laser itself, we tested the temperature change of pure water upon 10 min laser irradiation (Figures S32 and S33), which amounted to only a 2.5 °C increase. Furthermore, a cyclic temperature change was achieved upon cyclic laser irradiation. No significant change was observed. Most importantly, after five cycles of heating and cooling, the PTA-Ps could still heat up to the same maximum temperature as the first round (Figure 5f). Additionally, no absorption change was noticed for the PTA-Ps aqueous solution after laser irradiation, as shown in Figure 5g. Besides the photothermal properties, the morphological stability was tested as well using DLS and Cryo-TEM. The size, membrane structure, and spherical shape were still unchanged after laser irradiation (Figure 5h,i and Figure S34).

**Figure 6:**
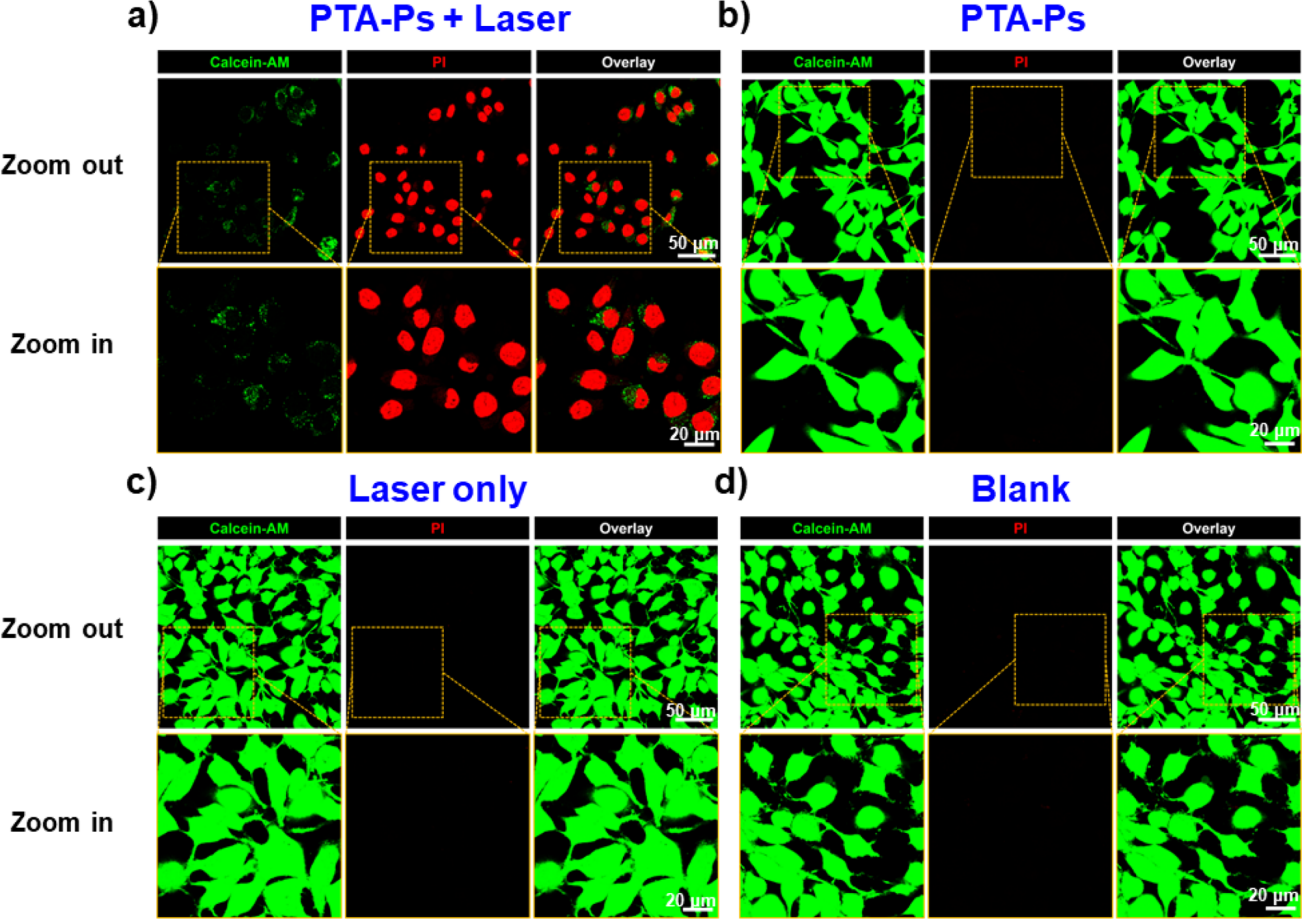
CLSM images of HeLa cells treated with different conditions (viable cells: calcein-AM, green; apoptotic cells: PI, red). (a) PTA-Ps with 808 nm laser irradiation with an output power of 0.53 W/cm^2^ for 20 min. (b) PTA-Ps without laser irradiation. (c) 808 nm laser irradiation with an output power of 0.53 W/cm^2^ for 20 min, in the absence of polymersomes. (d) Blank group (without laser irradiation and PTA-Ps). Scale bar: zoom out = 50 μm, zoom in = 20 μm.

Next, we investigated the therapeutic effect of PTA-Ps toward HeLa cells via PTT. First, we evaluated the cytotoxicity of PTA-Ps using the CCK-8 assay. As illustrated in Figure S35, the cell viability of HeLa cells remained more than 90% after incubation with PTA-Ps for 24 h, even at a high concentration of 200 μg/mL. The high cell viability suggests that PTA-Ps are biocompatible and have negligible dark cytotoxicity. Subsequently, cellular uptake of the PTA-Ps was evaluated. To visualize the PTA-Ps with confocal laser scanning microscopy (CLSM), Cy5 was loaded in the PTA-Ps. After incubation with HeLa cells for 19 h, Cy5-loaded PTA-Ps were observed in the cytoplasm of HeLa cells, as shown in Figure S36. Thereafter, the PTT effect of PTA-Ps was evaluated. HeLa cells were co-incubated with PTA-Ps (200 μg/mL) and then subjected to 808 nm laser irradiation (0.53 W/cm^2^). After irradiation for 20 min, dead cells were detected (Figure 6a). Furthermore, three parallel control experiments were conducted, including HeLa cells with PTA-Ps in the absence of laser irradiation, HeLa cells with laser irradiation in the absence of polymersomes, and HeLa cells without any treatment. In all three cases, good cell viability was observed (Figure 6b–d).

## Conclusions

In summary, we designed and developed an organic small molecule (PTA) that was composed of molecular rotors and a D–D′–A–D′–D conjugated backbone. This photothermal moiety achieved good absorption in the NIR window and exhibited low fluorescence in solution. The PTA was subsequently introduced into an amphiphilic block copolymer PEG_44_-PTA_2_, which was self-assembled into photothermal-responsive polymer vesicles (PTA-Ps). The well-defined PTA-Ps exhibited good performance in photothermal conversion and photostability. A 1 mg/mL polymer vesicle dispersion was able to heat the aqueous medium to 56 °C. Most importantly, the photothermal performance, optical properties, and nanostructural morphology of PTA-Ps were not changed after laser irradiation. Moreover, efficient in vitro PTT was achieved. Overall, the low cytotoxicity, good photothermal performance, and loading capacity demonstrate the great potential of PTA-Ps for biomedical applications.

## Supporting Information Available

Detailed experimental conditions and methods; molecular synthesis details; PTA-Ps and PTA-NPs preparation; photothermal performance; photothermal stability; cargo-loaded PTA-Ps preparation; quantification of the dye concentration; encapsulation efficiency and loading efficiency; PCE calculation; computational methods; cell culture; cell viability; cell uptake; in vitro photothermal therapy; ^1^H NMR spectra; ^13^C NMR spectra; ^19^F NMR spectra; GPC analysis; MALDI-TOF-MS spectra; DFT-optimized geometries of PTA; emission spectra; optical image; DLS analysis; SEM images; absorbance–concentration calibration curve; UV–vis–NIR absorption spectra; heating and cooling curves; photothermal curves; CLSM images (PDF)

## Abbreviations

DFT: density functional theory
NIR: near-infrared
PTA-Ps: photothermal-responsive polymer vesicles/polymersomes
PTA: photothermal agent
TPE: tetraphenylethylene
ATQ: acrylate-substituted thiadiazoloquinoxaline
PTMC: poly(trimethylene carbonate)
ROP: ring-opening polymerization
DP: degree of polymerization
SEM: scanning electron microscopy
DLS: dynamic light scattering
Cryo-TEM: cryogenic transmission electron microscopy
ICT: intramolecular charge transfer
TICT: twisted intramolecular charge transfer
CLSM: confocal laser scanning microscope
CCK-8: cell counting kit-8
PTT: photothermal therapy
PTA-NPs: PTA nanoparticles
