# Supplementary material for: Polymer Vesicles with Integrated Photothermal Responsiveness
Source: J Am Chem Soc. 2023 Sep 4;145(36):20073–80. doi: 10.1021/jacs.3c07134 (PMC10510318; doi:10.1021/jacs.3c07134)
Supplement: Supplementary file 1 — ja3c07134_si_001.pdf [file ja3c07134_si_001.pdf]

Supporting Information

Polymer Vesicles with Integrated Photothermal Responsiveness

Yingtong Luo,[1] Hanglong Wu,[1] Xuan Zhou,[2] Jianhong Wang,[1] Süleyman Er,[2] Yudong Li,[1] Pascal L.W. Welzen,[1] Roy A.J.F. Oerlemans,[1] Loai K. E. A. Abdelmohsen*,[1] Jingxin Shao*,[1] and Jan C. M. van Hest*,[1]

[1]Bio-Organic Chemistry, Institute of Complex Molecular Systems (ICMS), Eindhoven University of Technology, P.O. Box 513, 5600MB Eindhoven, The Netherlands

[2]DIFFER - Dutch Institute for Fundamental Energy Research, De Zaale 20, 5612 AJ Eindhoven, The Netherlands

*Corresponding should be address to: L. K. E. A. Abdelmohsen@tue.nl; J.Shao@tue.nl; J.C.M.v.Hest@tue.nl

Contents

1. Materials

2. Instruments

3. Methods

3.1 Synthesis of photothermal agent (PTA) and photothermal block copolymer (PEG_{44}-PTA_2).

3.2 Preparation of photothermal responsive polymer vesicles (PTA-Ps).

3.3 Preparation of photothermal nanoparticles (PTA-NPs)

3.4 Photothermal performance and loading capacity of PTA-Ps.

3.5 Calculation of photothermal conversion efficiency.

3.6 Computational methods

3.7 Cell experiments

4. Supplementary schemes, figures and table

Scheme S1. Synthesis route of molecular rotors (TPE-PIN and OH-TPE-PIN).

Figure S1. ^{1}H NMR and ^{13}C NMR spectrum of TPE-Cl.

Figure S2. ^{1}H NMR and ^{13}C NMR spectrum of TPE-PIN.

Figure S3. ^{1}H NMR and ^{13}C NMR spectrum of OH-TPE-Cl.

Figure S4. ^{1}H NMR and ^{13}C NMR spectrum of OH-TPE-PIN.

Figure S5. ^{1}H NMR and ^{13}C NMR spectrum of Compound 1.

Figure S6. ^{1}H NMR and ^{13}C NMR spectrum of Compound 2.

Figure S7. ^{1}H NMR and ^{13}C NMR spectrum of Compound 3.

Figure S8. ^{1}H NMR and ^{13}C NMR spectrum of Compound 4.

Figure S9. ^{1}H NMR and ^{13}C NMR spectrum of PTA.

Figure S10. ^{1}H NMR and ^{13}C NMR spectrum of Compound 5.

Figure S11. ^{1}H NMR spectrum of PTA-Boc.

Figure S12. ^{1}H NMR and ^{13}C NMR spectrum of PTA-NH_2.

Figure S13. ^{1}H NMR spectrum of PEG_{44}-PTA_2.

Figure S14. Representative ^{19}F NMR spectrum of PEG_{44}-PTA_2 (top) and PEG_{44}-$PTMCP_2$ (down), indicating full conversion of the TMCP group after post-modification.

Figure S15. MALDI-TOF-MS spectrum of Compound 2.

Figure S16. MALDI-TOF-MS spectrum of Compound 3.

Figure S17. MALDI-TOF-MS spectrum of Compound 4.

Figure S18. MALDI-TOF-MS spectrum of PTA.

Figure S19. MALDI-TOF-MS spectrum of Compound 5.

Figure S20. MALDI-TOF-MS spectrum of PTA-Boc.

Figure S21. MALDI-TOF-MS spectrum of PTA- NH_2.

Figure S22. GPC trace of PEG_{44}-$PTMCP_2$ and PEG_{44}-PTA_2.

Table S1. GPC analysis of block copolymers.

Figure S23. The DFT-optimized ground state (S_0) and excited state (S_1) geometries of PTA.

Figure S24. Characterization of Cy7 loaded PTA-Ps.

Figure S25. a) The average size of Cy7 loaded PTA-Ps and PTA-Ps. b) SEM images of Cy7 loaded PTA-Ps.

Figure S26. Characterization of Sulfo-Cy5 loaded PTA-Ps.

Figure S27. Absorbance-concentration calibration curve.

Figure S28. Th e normalized UV−vis−NIR absorption spectra of PEG_{44}-PTA_2 in THF.

Figure S29. The average size of PTA-NPs measured by DLS.

Figure S30. Photothermal stability of PTA-NPs, five circles of heating−cooling (808 nm, 0.90 W/cm^2).

Figure S31. The time constant of PTA-NPs heat transfer from the system, calculated with the linear time data from the system cooling period versus the negative natural logarithm of the system driving force temperature.

Figure S32. Plots of temperature vs time of pure water during heating via laser irradiation (808 nm, 0.90 W/cm^2) and upon cooling (laser off).

Figure S33. The time constant of water heat transfer from the system, calculated with the linear time data from the cooling period.

Figure S34. The average size of PTA-Ps (with and without 808 nm laser irradiation for 10 min) measured by DLS.

Figure S35. Cytotoxicity test of PTA-Ps after incubation with HeLa cells for 24 h.

Figure S36. CLSM images of HeLa cells after incubation with Cy5 loaded PTA-Ps (200 ug/ml) for 19 h, followed by staining of the cell membrane and nucleus with WGA-AF488 and Hoechst 33342.

5. References

1. Materials

All reagents and solvents were purchased from commercial sources (Fluorochem, Biosolve Chimie, Fisher Scientific, Sigma-Aldrich, TCI Europe, ABCR GmbH, ThermoFisher and Rapp Polymers etc) and used without further purification. Solvents used for synthesis were purified and dried using standard techniques. Dialysis membranes were purchased from Spectra/Pro® (NWCO 12,000-14,000). Amicon Ultra-0.5 Centrifugal Filter Unit (0.5 mL, 10 kDa) was from Millipore. Live cell imaging solution, Wheat Germ Agglutinin, Alexa Fluo™ 488 conjugate (WGA-AF488), Hoechst 33342, calcein-AM, propidium iodide (PI), Dulbecco's modified eagle medium (DMEM), phosphate-buffered saline (PBS, pH 7.4), no mycoplasma fetal bovine serum (FBS), trypsin-EDTA and penicillin-streptomycin were purchased from ThermoFisher. Cell counting kit-8 (CCK-8) was obtained from Merck. Ultrapure Milli-Q water (18.2 MΩ) was produced by a Merck Millipore Q-Pod system with a Millipore Express 40 filter (0.22 μm), and used for all experiments in this work.

2. Instruments

Proton Nuclear magnetic resonance spectroscopy (^{1}H NMR)

All compounds were characterized by routine proton nuclear magnetic resonance (^{1}H NMR) (400 MHz) on a Bruker Avance 400 MHz Ultrashield™ spectrometer equipped with a Bruker Sample Case auto-sampler, using $CDCl_3$ as the solvent and TMS as the internal standard.

Gel permeation chromatography (GPC)

The molecular weights and dispersity of the block copolymer were characterized by using a Prominence-I GPC system (Shimadzu) with a PL gel 5 μm mixed D (Polymer Laboratories) and equipped with an RID-20A differential refractive index detector. Polystyrene standards were used for calibration. THF was used as an eluent with a flow rate of 1 mL/min.

Scanning electron microscopy (SEM)

Morphological characterization of the polymer vesicles was performed using SEM (FEI Quanta 200 3D FEG) at 5.00 kV voltage.

Cryogenic transmission electron microscopy (cryo-TEM)

Cryo-TEM was conducted on the TU/e CryoTitan equipped with a field-emission gun operating at 300 kV, an autoloader station and a post-column Gatan. To prepare the cryo-TEM samples, 3 μL sample solution was pipetted on the grid (Lacey carbon coated, R2/2, Cu, EM sciences) and blotted in a Vitrobot MARK IV at 100% humidity to remove the excess solution.

Dynamic light scattering measurements (DLS)

The hydrodynamic size of the polymersome samples was characterized by using a Zetasizer (model Nano ZSP, Malvern Instruments) equipped with a 633 nm He-Ne laser and an avalanche photodiode detector. To process and analyze the data, Zetasizer software was used.

UV-vis-NIR spectrophotometer

The absorbance of sample solutions was characterized by using a Cary 3500 UV-vis-NIR spectrophotometer (Agilent).

Thermometer

Real time temperature of sample solutions upon laser irradiation was measured by using a thermometer (CHAUVIN ARNOUX C.A 1823).

Infrared camera

Thermal imaging pictures of sample solutions were taken with a camera (FLIR E54).

Matrix Assisted Laser Desorption/Ionization Time-of-Flight Mass Spectrometry (MALDI-TOF-MS) spectrum

The m/z of the organic compounds was characterized using a Bruker autoflex™ speed MALDI-TOF.

Microplate Reader

Cell viability was evaluated via a microplate reader (Safire2, TECAN). The reaction progress was monitored via the fluorescent signal from fluorescein on the Spark® 10M microplate reader (TECAN).

Confocal Laser Scanning Microscopy (CLSM)

Fluorescent images were observed and recorded using a CLSM (Leica TCS SP8X).

3. Methods

3.1 Synthesis of photothermal agent (PTA) and photothermal block copolymer (PEG_{44}-PTA_2)

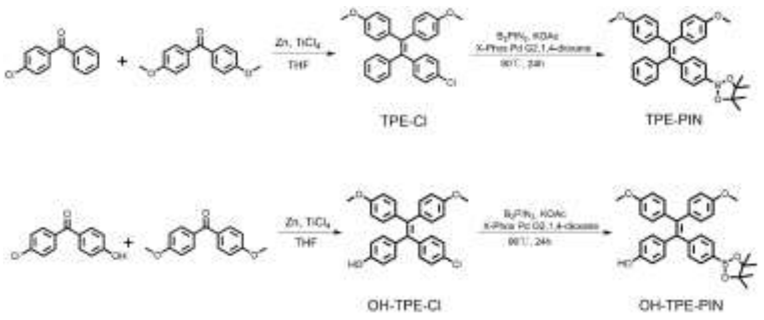

Scheme S1. Synthesis route of molecular rotors (TPE-PIN and OH-TPE-PIN)

TPE-Cl: This compound was synthesized according to the previously reported methods.[1] Bis(4-methoxyphenyl)methanone (9.73 g, 40.0 mmol), 4-chloro-benzophenone (10.4 g, 48.0 mmol), and Zn powder (13 g, 200 mmol) were dissolved in dry THF pre-cooled on an ice-bath (500 mL) under argon atmosphere. While stirring, titanium tetrachloride ($TiCl_4$, 17.6 mL, 160 mmol) was slowly added into the reaction mixture. Then the mixture was heated to 80 °C. After 24 h, the reaction mixture was left to cool down to room temperature. Subsequently, the reaction was quenched by the addition of 250 mL 10% aq K_2CO_3, and the mixture was filtered to remove insoluble materials and washed with CH_2Cl_2. The organic layer was dried with anhydrous $MgSO_4$ and filtered. The organic solvent was removed by evaporation. The residue was purified by silica gel chromatography with hexane/DCM (V_1:V_2 = 5/1) as the eluent to give 9.42 g of TPE-Cl as a yellow solid (7.9 g, 46% yield). ^{1}H NMR (400 MHz, $CDCl_3$) δ (ppm): 7.14-7.04 (m, 5H), 7.01-6.98 (m, 2H), 6.95-6.90 (m, 6H), 6.67-6.61 (m, 4H), 3.76 (d, J = 8 Hz, 6H); ^{13}C NMR (100 MHz, $CDCl_3$) δ (ppm): 158.29, 158.21, 132.72, 132.58, 132.56, 131.36, 127.94, 127.84, 113.22, 113.06, 55.14, 55.11.

TPE-PIN: This compound was synthesized according to previously reported methods with slight modifications.[1] Under a nitrogen atmosphere, TPE-Cl (426mg, 1 mmol), bis(pinacolato)diboron (B_2Pin_2) (1.53 g, 6 mmol), X-Phos Pd G2 pre-catalyst (40 mg, 0.05 mmol) and potassium acetate (KOAc) (590 mg, 6 mmol) were dissolved in anhydrous 1,4-dioxane (5 mL). The resulting mixture was heated to 90 °C and stirred for 24 hours. After cooling to room temperature, the reaction mixture was poured into 50 mL water and extracted with EtOAc (50 mL). The combined organic phase was dried by anhydrous magnesium sulfate, filtered and concentrated under vacuum. The crude product was used for the next synthesis step without further purification ^{1}H NMR (400 MHz, $CDCl_3$) δ (ppm): 7.55 (d, J = 8 Hz, 2H), 7.10- 6.98 (m, 7H), 6.94-6.92 (m, 4H), 6.63 (d, J = 8 Hz, 4H), 3.73 (d, J = 4 Hz, 6H), 1.32 (s, 12H); ^{13}C NMR (100 MHz, $CDCl_3$) δ (ppm):158.13, 158.10, 147.41, 144.13, 139.11, 136.29, 136.18, 134.10, 132.59, 131.40, 130.77, 127.66, 126.09, 113.06, 112.97, 83.66, 83.50, 55.05, 25.03, 24.90..

OH-TPE-Cl: Synthesized according to the TPE-Cl synthesis method (7.1 g, 40% yield). ^{1}H NMR (400 MHz, $CDCl_3$) δ (ppm): 7.07 (d, J = 8 Hz, 2H), 6.95- 6.85 (m, 8H), 6.64-6.63 (d, J = 8 Hz, 4H), 6.58 (d, J = 8 Hz, 2H), 4.70 (s, 1H), 3.75 (d, J = 4 Hz, 6H); ^{13}C NMR (100 MHz, $CDCl_3$) δ (ppm):158.12, 158.04, 154.00, 142.97, 139.95, 137.45, 136.43, 136.35, 136.18, 132.72, 132.56, 132.51, 131.73, 127.89, 114.77, 113.19, 113.11, 55.14.

OH-TPE-PIN: Synthesized according to the TPE-PIN synthesis method. The crude product was used for the next synthesis step without further purification. ^{1}H NMR (400 MHz, $CDCl_3$) δ (ppm): 7.54 (d, J = 8 Hz, 2H),7.03 (d, J = 8 Hz, 2H), 6.95-6.90 (m, 4H), 6.85 (d, J = 8 Hz, 2H), 6.65-6.60 (m, 4H), 6.56 (d, J = 8 Hz, 2H), 5.11 (s, 1H), 3.74 (d, J = 4 Hz, 6H), 1.32 (s, 12H); ^{13}C NMR (100 MHz, $CDCl_3$) δ (ppm): 158.02, 157.98, 153.88, 147.61, 139.80, 138.64, 136.69, 136.59, 136.36, 134.09, 132.77, 132.57, 130.82, 114.65, 113.06, 113.05, 83.73, 55.11, 55.06, 25.03, 24.90.

Compound 1: was synthesized according to the previously reported method.[2]

Compound 2: Compound 1 (518 mg, 1 mmol) and stannane, (4-dodecyl-2-thienyl) trimethyl (1.04 g, 2.5 mmol) were dissolved in chlorobenzene (5 mL) under a nitrogen atmosphere. The resulting mixture was heated to 110 °C and stirred for 48 hours. After cooling to room temperature, the reaction mixture was poured into 30 mL water and extracted with dichloromethane three times. The combined organic phases were dried by anhydrous magnesium sulfate, filtered and concentrated under vacuum. The residue was purified by silica gel chromatography with hexane/dichloromethane (V_1:V_2 = 3/1) as the eluent. The product was a dark blue solid (620 mg, yield: 70%). ^{1}H NMR (400 MHz, $CDCl_3$): δ (ppm) = 8.80 (s, 2H), 7.34 (s, 2H), 5.50-5.42 (m, 2H), 2.75-2.71 (m, 4H), 1.78-1.69 (m, 4H), 1.53 (d, J = 8 Hz, 12H), 1.44-1.26 (m, 36H), 0.89-0.86 (m, 6H); ^{13}C NMR (100 MHz, $CDCl_3$) δ (ppm): 163.68, 152.33, 143.39, 143.11, 135.94, 135.41, 134.07, 128.34, 122.63, 70.76, 31.93, 30.67, 29.71, 29.67, 29.55, 29.44, 29.37, 22.70, 21.91, 14.13; MALDI TOF-MS: m/z calculated for [M]$^+$ $C_{48}H_{68}N_4O_4S_3$, 860.440, found 860.440.

Compound 3: Compound 2 (500 mg, 0.58 mmol) was dissolved in a mixture of 12 mL $CHCl_3$ and 6 mL acetic acid under argon atmosphere, NBS (206 mg, 1.16 mmol) was added slowly over the course of 30 min under the exclusion of light. The resulting mixture was stirred for 12 hours at room temperature. Then, the reaction mixture was poured into 30 mL water and extracted with dichloromethane three times. The combined organic phases were dried by anhydrous magnesium sulfate, filtered and concentrated under vacuum. The residue was purified by silica gel chromatography with hexane/dichloromethane (V_1:V_2 = 3/1) as the eluent. The product was a light green solid (472 mg, yield: 80%). ^{1}H NMR (400 MHz, $CDCl_3$): δ (ppm) = 8.68 (s, 2H), 5.53-5.44 (m, 2H), 2.62-2.58 (m, 4H), 1.67-1.64 (m, 4H), 1.59 (d, J = 8 Hz, 12H), 1.37-1.26 (m, 36H), 0.89-0.85 (m, 6H); ^{13}C NMR (100 MHz, $CDCl_3$) δ (ppm): 163.25, 151.62, 142.62, 142.04, 135.34, 135.04, 133.26, 121.11, 120.50, 71.03, 31.95, 29.73, 29.70, 29.69, 29.52, 29.42, 29.39, 22.71, 22.05, 14.14; MALDI TOF-MS: m/z calculated for [M]$^+$ $C_{48}H_{66}Br_2N_4O_4S_3$, 1018.26, found 1018.25.

Compound 4: Compound 3 (1630 mg, 1.6 mmol), TPE-PIN (427 mg, 0.8 mmol), potassium carbonate (1100 mg, 8 mmol), THF (48 mL)/water (16 mL), and $Pd(PPh_3)_4$ (5 %), were added into a flask under argon atmosphere. The reaction mixture was stirred at 60 °C for 12 h. After cooling down the reaction mixture to ambient temperature, it was extracted with dichloromethane and washed with water. The dichloromethane layer was separated and dried over Na_2SO_4. After dichloromethane evaporation by rotary evaporation, the crude product was purified by column chromatography on silica gel using n-hexane/dichloromethane (V_1:V_2 = 3/1) as the eluent. The product was a dark green solid (638 mg, yield: 30%). ^{1}H NMR (400 MHz, $CDCl_3$): δ (ppm) = 8.99 (s, 1H), 8.88 (s, 1H), 7.34-7.32 (d, J = 8 Hz, 2H), 7.17-7.08 (m, 7H), 7.10-6.95 (m, 4H), 6.68-6.64 (t, 4H), 5.52-5.39 (m, 2H), 3.75 (s, 6H), 2.78-2.68 (m, 4H), 1.76-1.66 (m, 4H), 1.58-1.56 (d, J = 8 Hz, 6H), 1.45-1.43 (d, J = 8 Hz, 6H), 1.42-1.20 (m, 36H), 0.88-0.85 (t, 6H); ^{13}C NMR (100 MHz, $CDCl_3$) δ (ppm): 158.28, 158.17, 132.71, 132.66, 131.58, 131.53, 128.26, 127.78, 113.12, 113.05, 70.98, 70.77, 55.12, 55.08, 31.95, 31.00, 29.89, 29.76, 29.72, 29.70, 29.68, 29.64, 29.59, 29.54, 29.52, 29.39, 22.72, 22.03, 21.91, 14.15; MALDI TOF-MS: m/z calculated for [M]$^+$ $C_{76}H_{89}N_4O_6S_3$, 1330.511, found 1330.53.

PTA: Compound 3 (300 mg, 0.3 mmol), TPE-PIN (393 mg, 0.76 mmol), potassium carbonate (324 mg, 2.36 mmol), and $Pd(PPh_3)_4$ (5%) were dissolved in a solution containing 9 mL THF and 3 mL water under argon atmosphere. The reaction mixture was heated to 60 °C and stirred for 12 h. After cooling to room temperature, the reaction mixture was extracted with dichloromethane and washed with water. The combined organic phases were dried using anhydrous $MgSO_4$, followed by filtering and concentrating under vacuum. The residue was purified by silica gel chromatography using hexane/dichloromethane (V_1:V_2 = 1:1) as the eluent. The obtained compound was a dark green solid (295 mg, 60% yield). ^{1}H NMR (400 MHz, $CDCl_3$): δ (ppm) = 8.98 (s, 2H), 7.35 (d, 4H), 7.11-7.08 (m, 10H), 7.01-6.95 (m, 10H), 6.68-6.64 (m, 10H), 5.46-5.38 (m, 2H), 3.75 (s, 12H), 2.80-2.74 (m, 4H), 1.75-1.67 (m, 4H), 1.45 (d, 12H), 1.37-1.25 (m, 36H), 0.89-0.85 (m, 6H); ^{13}C NMR (100 MHz, $CDCl_3$) δ (ppm): 163.77, 158.27, 158.16, 152.37, 144.17, 143.89, 142.84, 140.61, 139.24, 138.73, 136.34, 132.70, 132.65, 132.38, 131.57, 131.56, 131.53, 128.29, 127.76, 126.22, 113.11, 113.04, 70.71, 55.11, 55.08, 31.94, 31.01, 29.74, 29.69, 29.61, 29.53, 29.39, 28.94, 22.71, 21.89, 14.14; MALDI TOF-MS: m/z calculated for [M]$^+$ $C_{104}H_{112}N_4O_8S_3$, 1641.76, found 1641.76.

Compound 5: Compound 4 (1000 mg, 0.75 mmol), OH-TPE-PIN (480 mg, 0.9 mmol), potassium carbonate (830 mg, 6 mmol), THF (15 mL)/water (5 mL), and $Pd(PPh_3)_4$ (5 %), were added into a flask under argon atmosphere. The reaction mixture was stirred at 60 °C for 12 h. After cooling down the reaction mixture to ambient temperature, it was extracted with dichloromethane and washed with water. The dichloromethane layer was separated and dried over Na_2SO_4. After dichloromethane evaporation by rotary evaporation, the crude product was purified by column chromatography on silica gel using n-hexane/dichloromethane (V_1:V_2=2/1) as the eluent. The product was a dark green solid (745 mg, yield: 60%). ^{1}H NMR (400 MHz, $CDCl_3$): δ (ppm) = 8.98 (s, 2H), 7.34-7.33 (d, J = 4 Hz, 4H), 7.15-7.08 (m, 9H), 7.10-6.95 (m, 10H), 6.68-6.60(t, 10H), 5.44-5.36 (m, 2H), 4.62 (s, 1H), 3.77-3.74 (t, 12H), 2.79-2.73 (m, 4H), 1.78-1.67 (m, 4H), 1.45-1.43 (d, J = 8 Hz, 12H), 1.38-1.20 (m, 36H), 0.88-0.85 (t, 6H); ^{13}C NMR (100 MHz, $CDCl_3$) δ (ppm): 163.80, 158.15, 152.36, 134.11, 132.86, 132.62, 131.57, 131.53, 128.30, 128.27, 127.77, 113.10, 113.05, 55.13, 55.08, 31.95, 31.02, 29.76, 29.70, 29.63, 29.54, 29.40, 28.95, 22.72, 21.91, 14.15; MALDI TOF-MS: m/z calculated for $[M]^+$ $C_{104}H_{112}N_4O_9S_3$, 1657.762, found 1657.78.

PTA-Boc: A mixture of compound 5 (166 mg, 0.1 mmol), 6-(Boc-amino)hexyl bromide (28 mg, 0.1 mmol), and K_2CO_3 (34 mg, 0.25 mmol) in DMF (5 mL) was stirred at 80 °C overnight under argon atmosphere. The reaction mixture was cooled to room temperature and filtered through a pad of celite, washed with CH_2Cl_2. The solution was then dried with Na_2SO_4 and filtered. The solvent was removed by evaporation. The crude product was purified by column chromatography on silica gel using n-hexane/dichloromethane (V_1:V_2=1/1) as the eluent. The product was a dark green solid (56 mg, yield: 30%). ^{1}H NMR (400 MHz, $CDCl_3$): δ (ppm) = 8.98 (s, 2H), 7.35-7.33 (d, 4H), 7.17-7.07 (m, 9H), 7.02-6.94 (m, 10H), 6.71-6.61(m, 10H), 5.45-5.39 (m, 2H), 3.94-3.87 (m, 2H), 3.77-3.74 (m, 12H), 3.50-3.48 (m, 2H), 3.15-3.10 (m, 2H), 2.79-2.75 (m, 4H), 1.79-1.68 (m, 6H), 1.45-1.43 (m, 22H), 1.34-1.21 (m, 36H), 0.88-0.85 (t, 6H); MALDI TOF-MS: m/z calculated for $[M]^+$ $C_{115}H_{133}N_5O_{11}S_3$, 1856.92, found 1856.92.

PTA-NH_2: PTA-Boc (370 mg, 0.2 mmol) was dissolved in DCM (1 mL) and immersed in an ice bath. Then, 0.5 mL trifluoroacetic acid was added dropwise under nitrogen atmosphere. The reaction mixture was allowed to warm to room temperature and reacted for another 1 h. The dichloromethane layer was separated and dried over Na_2SO_4. After dichloromethane evaporation by rotary evaporation, the crude product was purified by column chromatography on silica gel using MeOH/dichloromethane (V_1:V_2=1/20) as the eluent. The product was a dark green solid (281 mg, yield: 80%). ^{1}H NMR (400 MHz, $CDCl_3$): δ (ppm) = 8.91 (s, 2H), 7.33-7.30 (m, 4H), 7.15-7.07 (m, 9H), 7.01-6.94 (m, 10H), 6.69-6.62 (m, 10H), 5.45-5.38 (m, 2H), 3.91-3.87 (m, 2H), 3.75-3.73 (m, 12H), 2.96 (s, 2H), 2.77-2.70 (m, 4H), 1.45-1.42 (m, 12H), 1.37-1.21 (m, 36H), 0.89-0.82 (t, 6H); ^{13}C NMR (100 MHz, $CDCl_3$) δ (ppm): 158.26, 158.15, 144.19, 138.75, 134.02, 132.70, 131.54, 128.29, 127.76, 113.12, 113.04, 70.67, 55.11, 55.07, 55.04, 31.95, 31.00, 29.75, 29.72, 29.69, 29.64, 29.54, 29.39, 29.15, 28.93, 25.65, 22.71, 21.91, 14.15; MALDI TOF-MS: m/z calculated for $[M]^+$ $C_{110}H_{125}N_5O_9S_3$, 1756.867, found 1756.87.

Block copolymer (PEG_{44}-PTA_2): PEG_{44}-$PTMCP_2$ was synthesized according to a previously reported method.[3] PEG_{44}-$PTMCP_2$ (52 mg, 0.02 mmol) was dissolved in 1 mL dry THF and cooled on an ice bath. Next, a 0.5 mL THF solution containing PTA-NH_2 (88 mg, 0.05 mmol, 1.15 equiv. with respect to the pentafluorophenyl ester) and trimethylamine (TEA, 10 μL, 0.05 mmol, 1.15 equiv. with respect to the pentafluorophenyl ester) were dropwise added. The ice bath was removed and the mixture was allowed to stir for an additional 90 min. The reaction was monitored by ^{1}HNMR and ^{19}F NMR spectroscopy. After the complete conversion of the pentafluorophenyl ester, the reaction solution was precipitated into diethyl ether three times. Then the green solid was isolated and freeze dried to obtain PEG_{44}-PTA_2 (64 mg, yield: 56%). ^{1}H NMR (400 MHz, $CDCl_3$): δ (ppm) = 8.97 (s, 4H), 7.32-7.34 (m, 8H), 7.08-7.15 (m, 18H), 6.95-6.99 (m, 20H), 6.64-6.68 (m, 20H), 5.39-5.45 (m, 4H), 3.86-3.92 (m, 4H), 3.71-3.77 (m, 24H), the protons of PEG 3.61-3.66 (m, 176H), terminal methyl unit 3.38 (s, 3H), 2.74-2.79 (m, 8H), 1.43-1.45 (d, J = 8 Hz, 24H), 1.21-1.32 (m, 72H), 0.86-0.88 (m, 12H). GPC (RI): Mn (PDI) = 5.73 kDa (1.09).

3.2 Preparation of photothermal responsive polymer vesicles (PTA-Ps)

First, 1mg PEG_{44}-PTA_2 was dissolved in 0.5 mL THF solution in a glass vial with a magnetic stirring bar. After stirring for 10 min, 0.5 mL of ultrapure Milli-Q water was added via a syringe pump (Chemyx, Inc., Fusion 100, KR Analytical Limited, Stafford, TX, USA) with a speed of 0.25 mL/h. The resulting cloudy solution was transferred into a 2 mL/cm pre-hydrated dialysis bag (MWCO 12,000-14,000, Spectra/Pro®, Rancho Dominguez, CA, USA) for dialysis against Milli-Q water at 4 °C with a water change after 1 h, followed by dialyzing for at least 24 h.[3] (The membrane thickness was determined from Cryo TEM images by analysis of 30 different assemblies)

3.3 Preparation of photothermal nanoparticles (PTA-NPs)

Amphiphilic block polymer (PEG_{44}-$PTMC_{100}$) was synthesized according to a previously reported method.[3] A THF solution (1 mL) containing PEG_{44}-$PTMC_{100}$ (10 mg), PTA (1.0 mg) and Milli-Q water (10.0 mL) were emulsified by sonication for 10 min. Then the solution was vacuum concentrated to evaporate the THF and then purified by being passed through a membrane filter (diameter = 220 nm).[2]

3.4 Photothermal performance and loading capacity of PTA-Ps

Photothermal performance: Samples (PTA and PTA-Ps) were continuously irradiated with an 808 nm NIR laser for 10 min. The temperature was measured every second using a digital thermometer with a thermocouple probe. The IR thermal images of the sample tubes were also recorded.[4]

Photothermal stability: For photostability and morphology stability studies, the PTA-Ps solution was irradiated with an 808 nm laser for 10 min, and the absorption spectra and Cryo-TEM image were measured. For photothermal stability studies, the temperatures of the sample solutions were recorded during five circles of heating and cooling. In each heating-cooling circle, the NIR laser was first used to irradiate the samples for 10 min, then the laser was turned off, and the samples were naturally cooled down for 10 min.

Preparation of Cy7 loaded PTA-Ps: First, 1mg PEG_{44}-PTA_2 and 0.1 mg Cy7-NHS ester were dissolved in 0.5 mL THF solution in a glass vial with a magnetic stirring bar. After stirring for 10 min, 0.5 mL of ultrapure Milli-Q water was added via a syringe pump (Chemyx, Inc., Fusion 100, KR Analytical Limited, Stafford, TX, USA) with a speed of 0.25 mL/h. The resulting cloudy solution was transferred into a 2 mL/cm pre-hydrated dialysis bag (MWCO 12,000-14,000, Spectra/Pro®, Rancho Dominguez, CA, USA) for dialysis against Milli-Q water at 4 °C with a water change after 1 h, followed by dialyzing for at least 24 h.

Preparation of Cy5 loaded PTA-Ps: First, 1mg PEG_{44}-PTA_2 was dissolved in 0.5 mL THF solution in a glass vial with a magnetic stirring bar. After stirring for 10 min, 0.5 mL of ultrapure Milli-Q water containing 0.05 mg Sulfo-Cy5-NHS ester was added via a syringe pump (Chemyx, Inc., Fusion 100, KR Analytical Limited, Stafford, TX, USA) with a speed of 0.25 mL/h. The resulting cloudy solution was transferred into a 2 mL/cm pre-hydrated dialysis bag (MWCO 12,000-14,000, Spectra/Pro®, Rancho Dominguez, CA, USA) for dialysis against Milli-Q water at 4 °C with a water change after 1 h, followed by dialyzing for at least 24 h.

Quantification of the Sulfo-Cy5 and Cy7 concentrations: The Sulfo-Cy5 and Cy7 concentrations were determined by measuring the absorbance at 648 and 745 nm, respectively. The control samples (empty PTA-Ps) were also measured, and their scattering contributions to the absorbance were subtracted from the cargo-loaded PTA-Ps formulations. Absorbance values were correlated with the Sulfo-Cy5 or Cy7 concentration based on calibration curves.[5]

Encapsulation efficiency and loading efficiency: The encapsulation efficiency (*EE*) and loading efficiency (*LE*) was calculated according to the reported method.[5] The detailed calculation was carried out using the following equations:

$$EE = \frac{m_e}{m_c}$$

$$LE = \frac{m_e}{m_t}$$

where m_e is the weight of encapsulated cargo, m_c is the total weight of cargo initially added in formulation and m_t is the total weight of copolymer and cargo.

3.5 Calculation of photothermal conversion efficiency

The photothermal conversion efficiency (η) of the PTA-Ps was calculated according to the reported method.[6] The detailed calculation was carried out using the following equations:

$$\eta = \frac{hA(T_{max} - T_{surr}) - Q_{dis}}{I(1 - 10^{-A_{808}})}$$

... (1)

$$hA = \frac{mC_{water}}{\tau_s}$$

... (2)

$$t = -\tau_s ln\theta = -\tau_s \ln(\frac{T - T_{surr}}{T_{max} - T_{surr}})$$

... (3)

$$Q_{dis} = \frac{mC_{water}(T_{\max(water)} - T_{surr})}{\tau_{water}}$$

... (4)

where h is the heat transfer coefficient, A is the surface area of the container, m is the mass of the solution (m = 0.5 g), Q_{dis} is measured independently to be 0.035 W, τ_s is a system time constant of 204.63 s, C_{water} = 4.2 J/g •°C, I is incident laser power (0.90 W/cm^2), η is the photothermal conversion efficiency, A_{808} indicates the absorbance of PTA-Ps solution (A_{808} = 2.63) , T_{surr} is the surrounding temperature, T_{max} and $T_{max(water)}$, are the max temperature of PTA-Ps solution and water, respectively. The PTA-Ps photothermal conversion efficiency (η/PCE) was calculated to be 31.4%. According to the same method, the η (or PCE) of PTA-NPs is calculated as 29.0%

3.6 Computational methods

All the simulations were performed in Schrödinger Materials Science Suite (SMSS).[7] The initial three-dimensional geometry of the PTA molecule was generated by using the Maestro editor. Then a conformational search was performed by using the OPLS3e force field (FF) to find the lowest-energy conformer of the PTA molecule.[8] The lowest energy conformer was further optimized using the density functional theory (DFT) calculations in the gas phase. The implicit solvation model with standard Poisson–Boltzmann Formalism (PBF) was applied for single point energy (SPE) calculations in water.[9] For the optimization of the excited state geometries, we used the time-dependent density functional theory (TD-DFT) calculations with full linear response formalism. The MacroModel module and Jaguar program, as both implemented in SMSS, were employed for FF and DFT/TD-DFT calculations, respectively.[10,11] The B3LYP functional and LACVP^{++**} basis set with polarization and diffuse functions were employed in both DFT and TD-DFT simulations.[12-14] The changes in energy and root mean square density matrix were set at 5×10^{-5} and 5×10^{-6} Hartree, respectively. For DFT and TD-DFT optimizations, the default settings of 'Medium' and 'Quick' were used for the grid density and accuracy level, respectively, whereas for SPE calculations, 'Fine' and 'Accurate' settings were used, respectively.

3.7 Cell experiments

Cell culture: Human cervical cancer cells (HeLa) were cultured in Dulbecco's Modified Eagle Medium (DMEM) containing 10% fetal bovine serum (FBS), 1% penicillin-streptomycin at 37 °C in a cell incubator (Thermo Fisher) under an atmosphere of 5% CO_2 and 70% humidity.

Cell viability in CCK-8 Assay: To evaluate the feasibility of the PTA-Ps for photothermal therapy, we performed a systematic evaluation of their in vitro cytotoxicity using a standard CCK-8 assay. HeLa cells were used as a tumor model. Briefly, the cells were seeded in 96 well plates at a density of 1×10^4 cells per well in 100 μL DMEM medium and cultured overnight. Then the medium was refreshed and PTA-Ps at different concentrations (0, 5, 10, 20, 40, 60, 80, 100, 200 μg/mL) were added. After 24 h incubation, the cells were washed three times with PBS and treated with 100 μL of DMEM containing 10% CCK-8 for each well for 3 h. The cell viability was calculated using the absorbance recorded at 450 nm by a microplate reader. For each group, triplicated wells were tested, and the data were presented as mean ± SD.

Cell uptake: To investigate the intracellular uptake of photothermal PTA-Ps, HeLa cells were incubated with Cy5 loaded PTA-Ps (200 μg/mL). After incubation for 19 h, the cells were washed three times with live cell imaging solution and stained with WGA-AF488 and Hoechst 33342. Subsequently, the samples were characterized using CLSM.

Evaluation in photothermal therapy of PTA-Ps: To investigate the therapeutic effect of PTA-Ps, HeLa cells were cultured with PTA-Ps (200 μg/mL). After incubation for 19 h, the cells were washed three times with PBS to remove the free PTA-Ps and refreshed with DMEM. Then the cells were irradiated with an 808 nm laser at 0.56 W/cm^2 for 20 min. After incubation for another 4 h, the cells were washed with live cell imaging solution and stained with calcein-AM and PI. Subsequently, the samples were characterized using CLSM. Three parallel experiments were designed as control groups, including HeLa cells with PTA-Ps in the absence of laser irradiation, HeLa cells with laser irradiation in absence of polymersomes, and HeLa cells without any treatment.

4. Supplementary figures

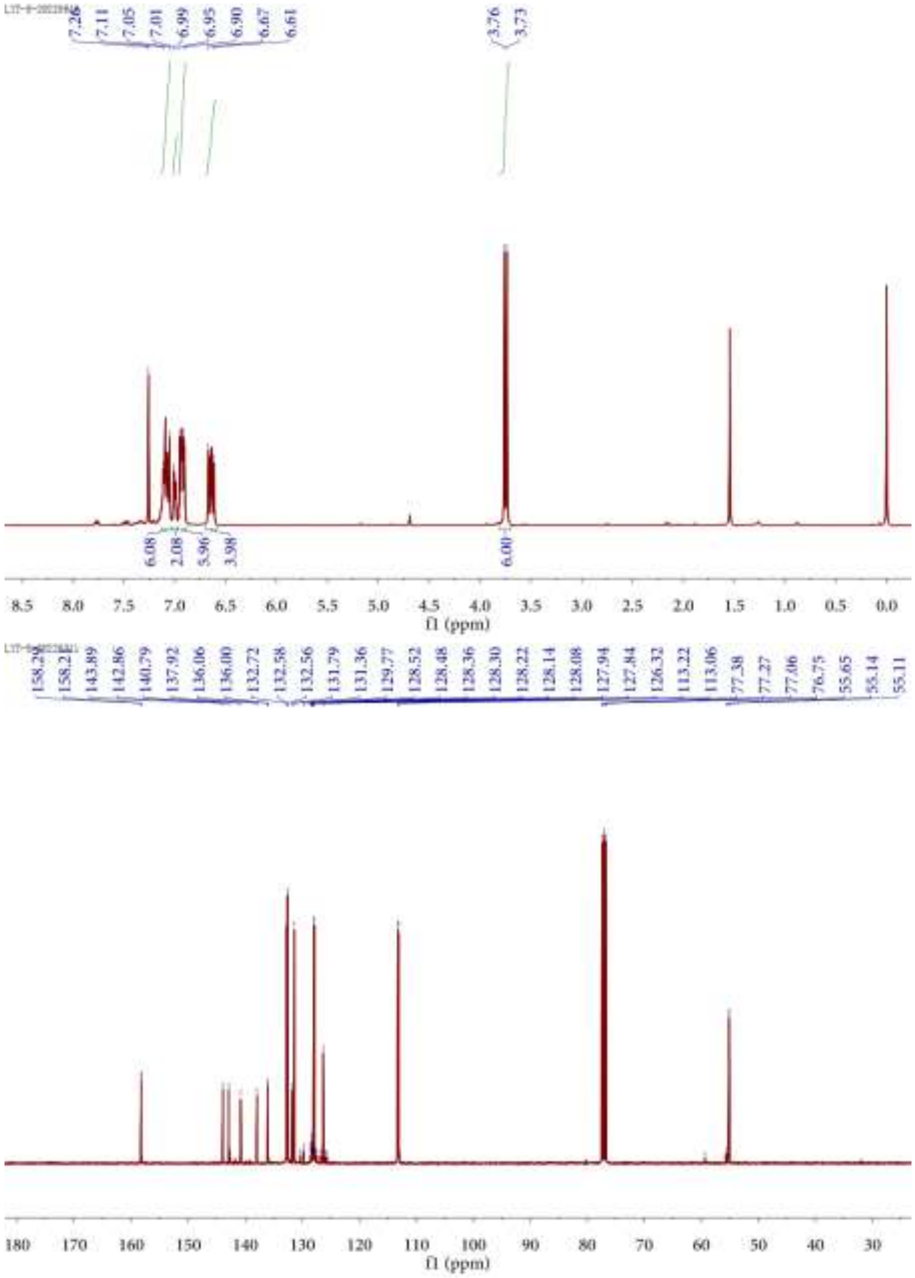

Figure S1. ^{1}H NMR and ^{13}C NMR spectrum of TPE-Cl (400 MHz, 298 K, $CDCl_3$).

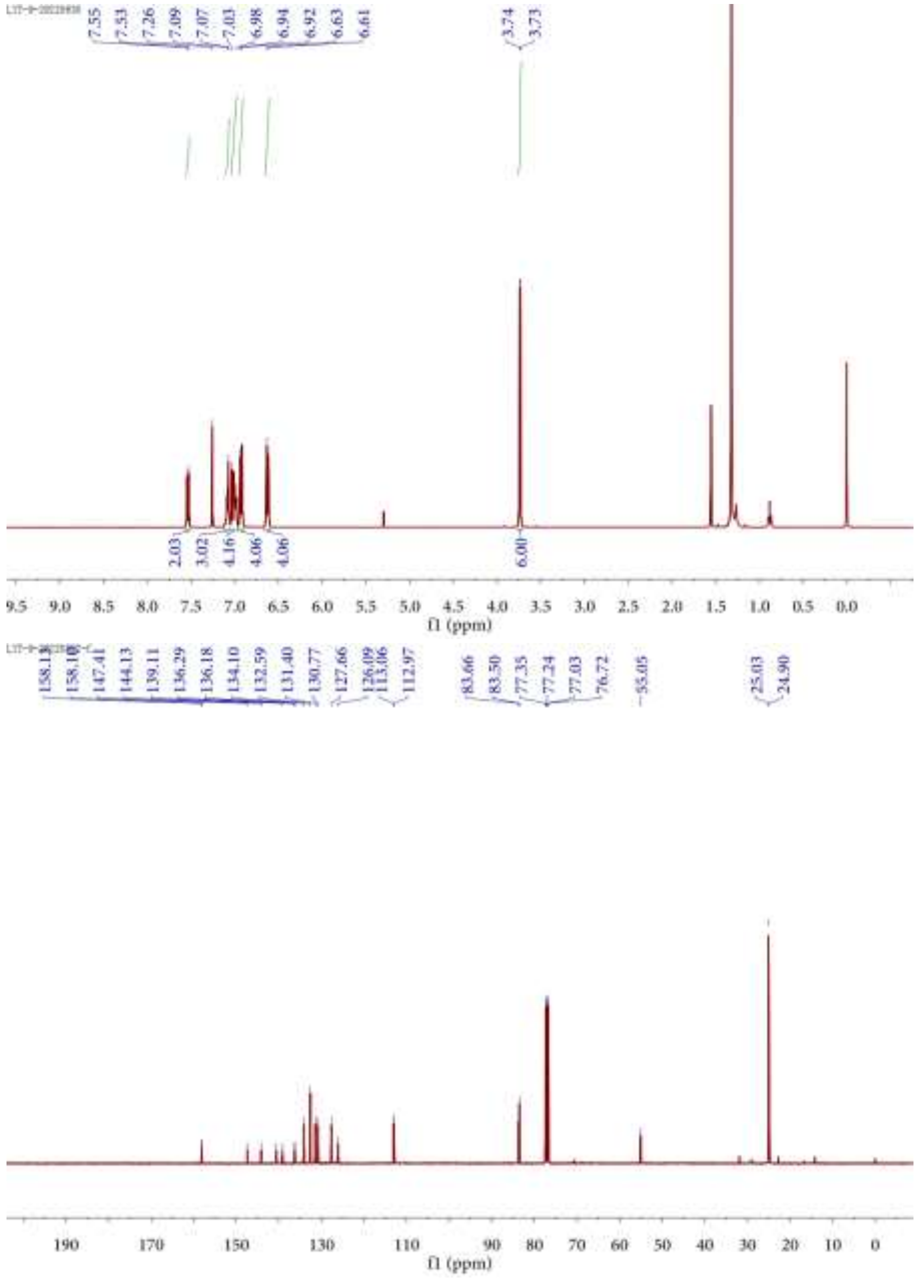

Figure S2. ^{1}H NMR and ^{13}C NMR spectrum of TPE-PIN (400 MHz, 298 K, $CDCl_3$).

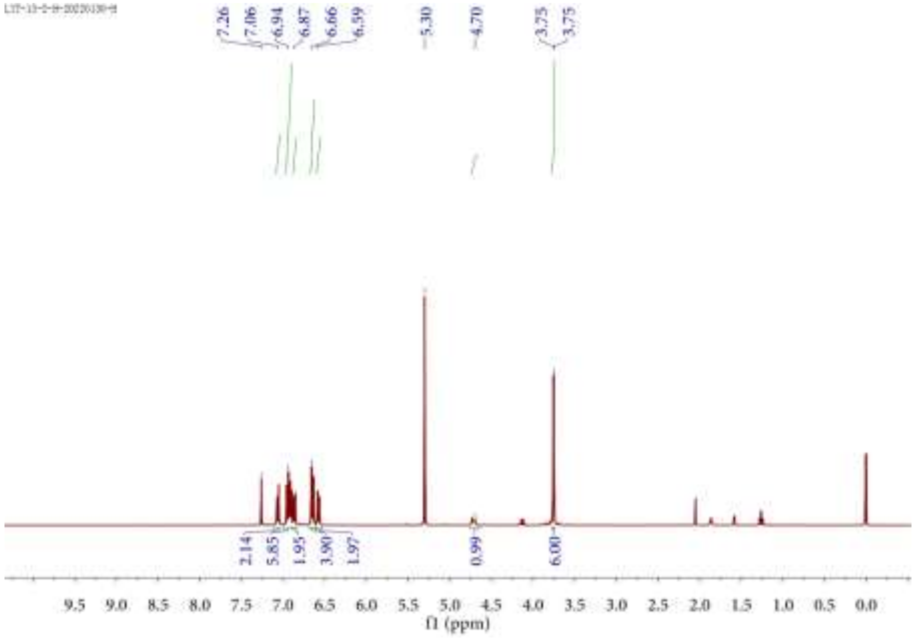

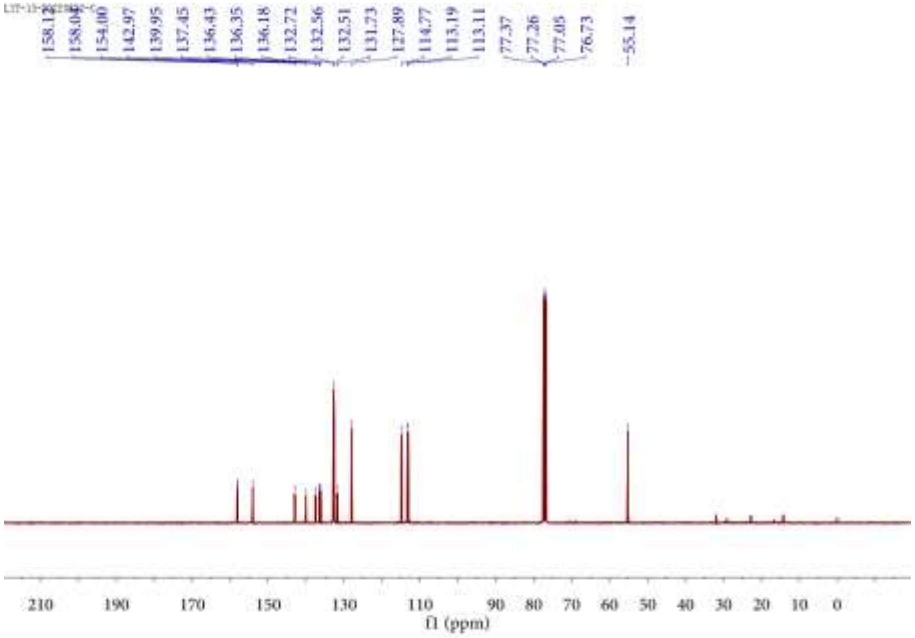

Figure S3. ^{1}H NMR and ^{13}C NMR spectrum of OH-TPE-Cl (400 MHz, 298 K, $CDCl_3$).

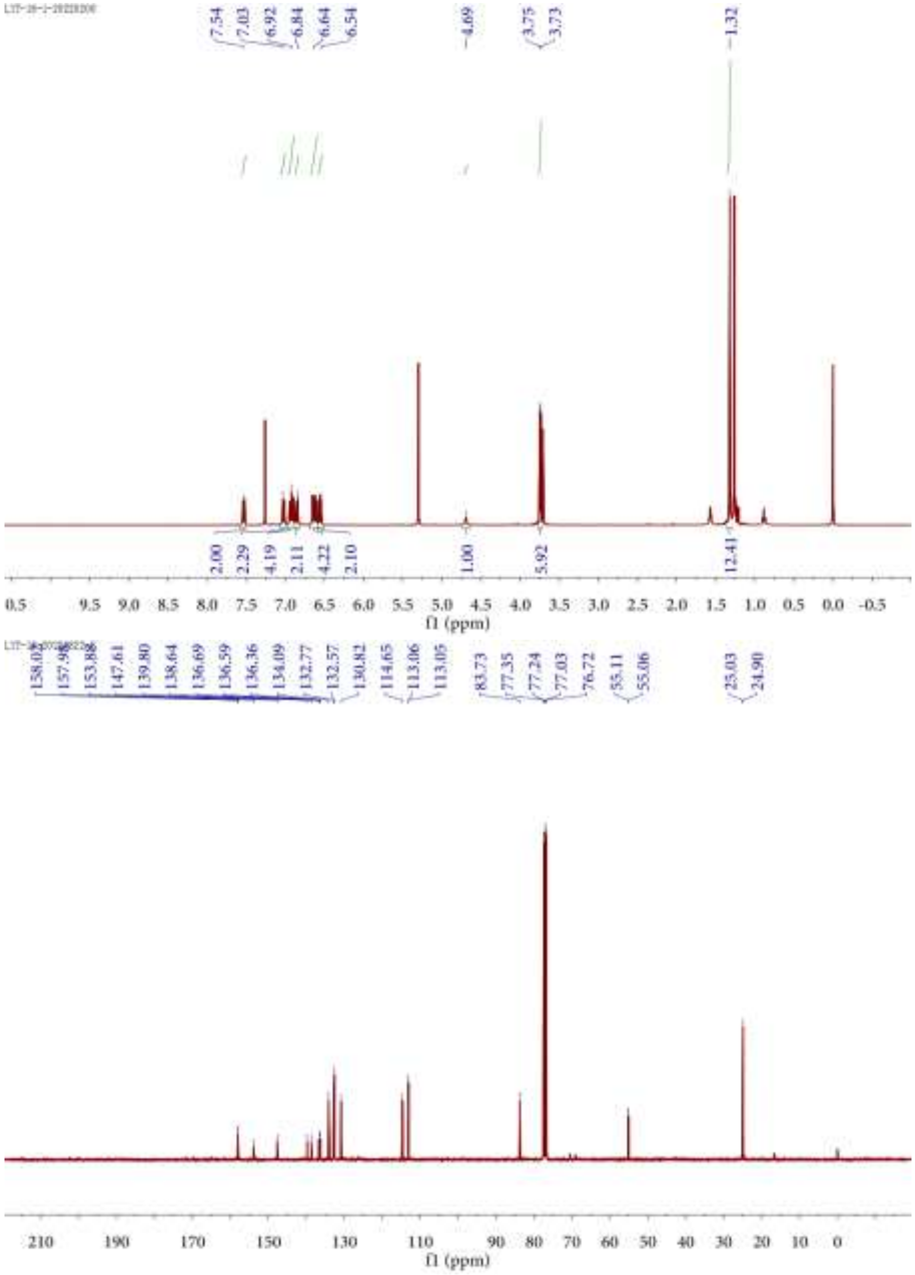

Figure S4. ^{1}H NMR and ^{13}C NMR spectrum of OH-TPE-PIN (400 MHz, 298 K, $CDCl_3$).

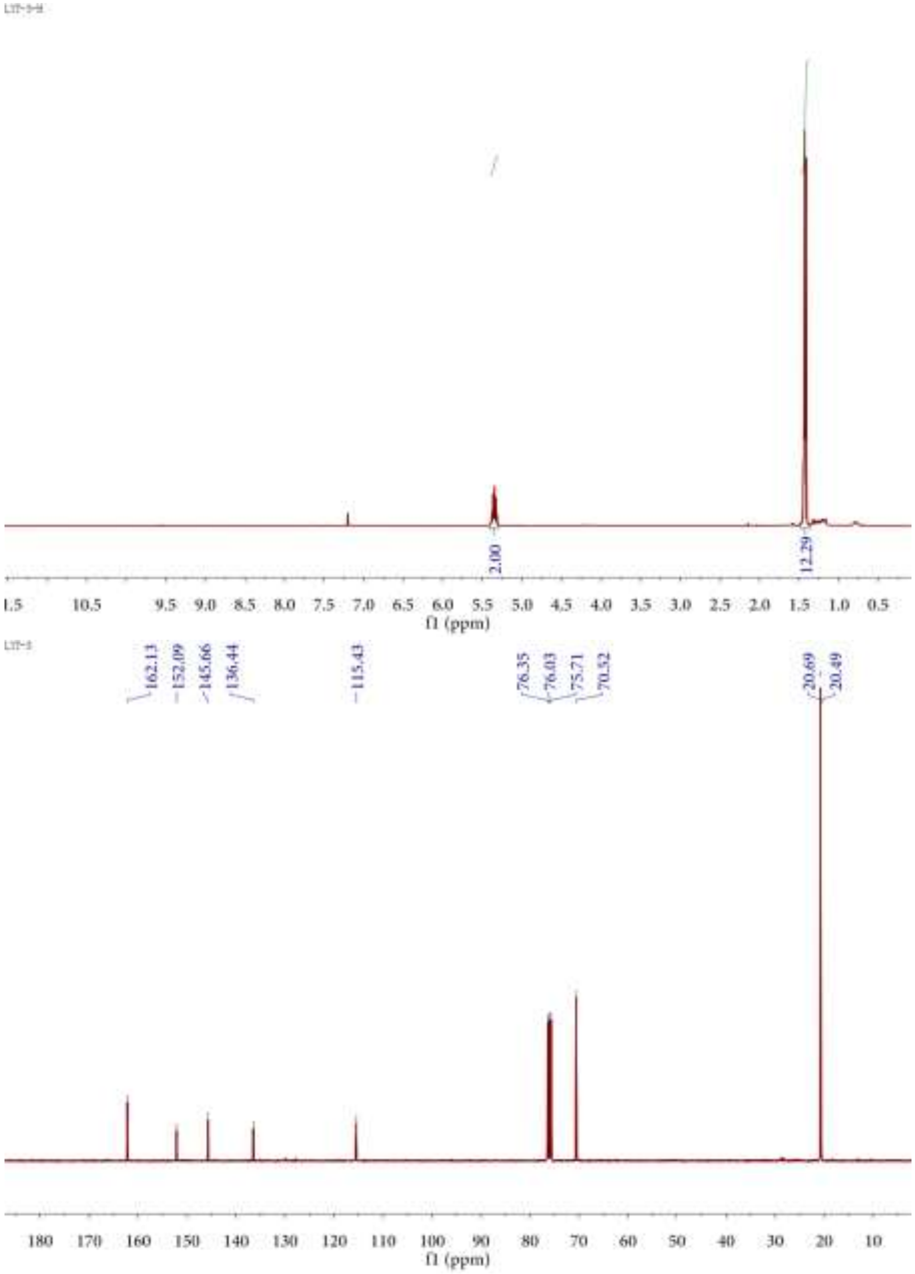

Figure S5. ^{1}H NMR and ^{13}C NMR spectrum of Compound 1 (400 MHz, 298 K, $CDCl_3$).

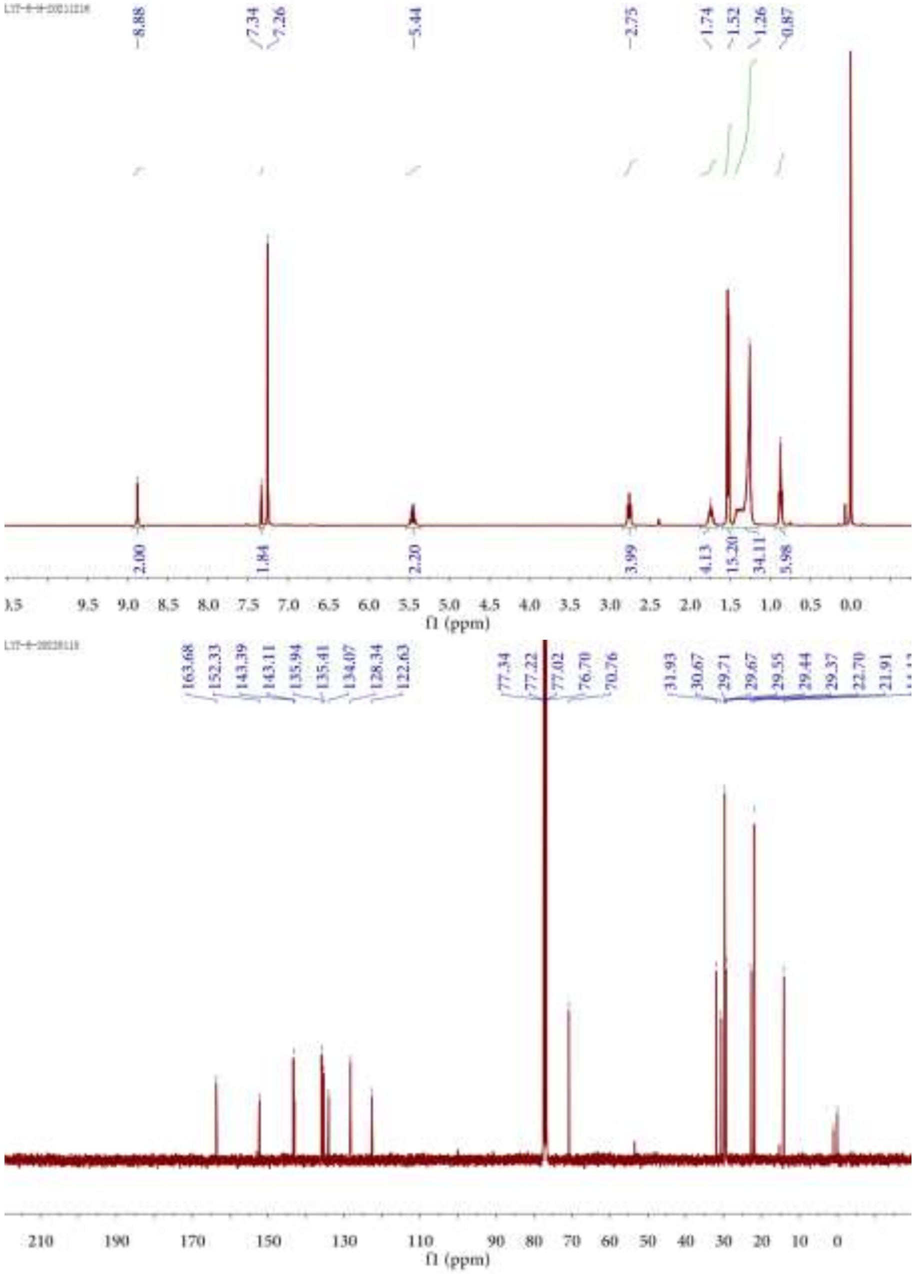

Figure S6. ^{1}H NMR and ^{13}C NMR spectrum of Compound 2 (400 MHz, 298 K, $CDCl_3$).

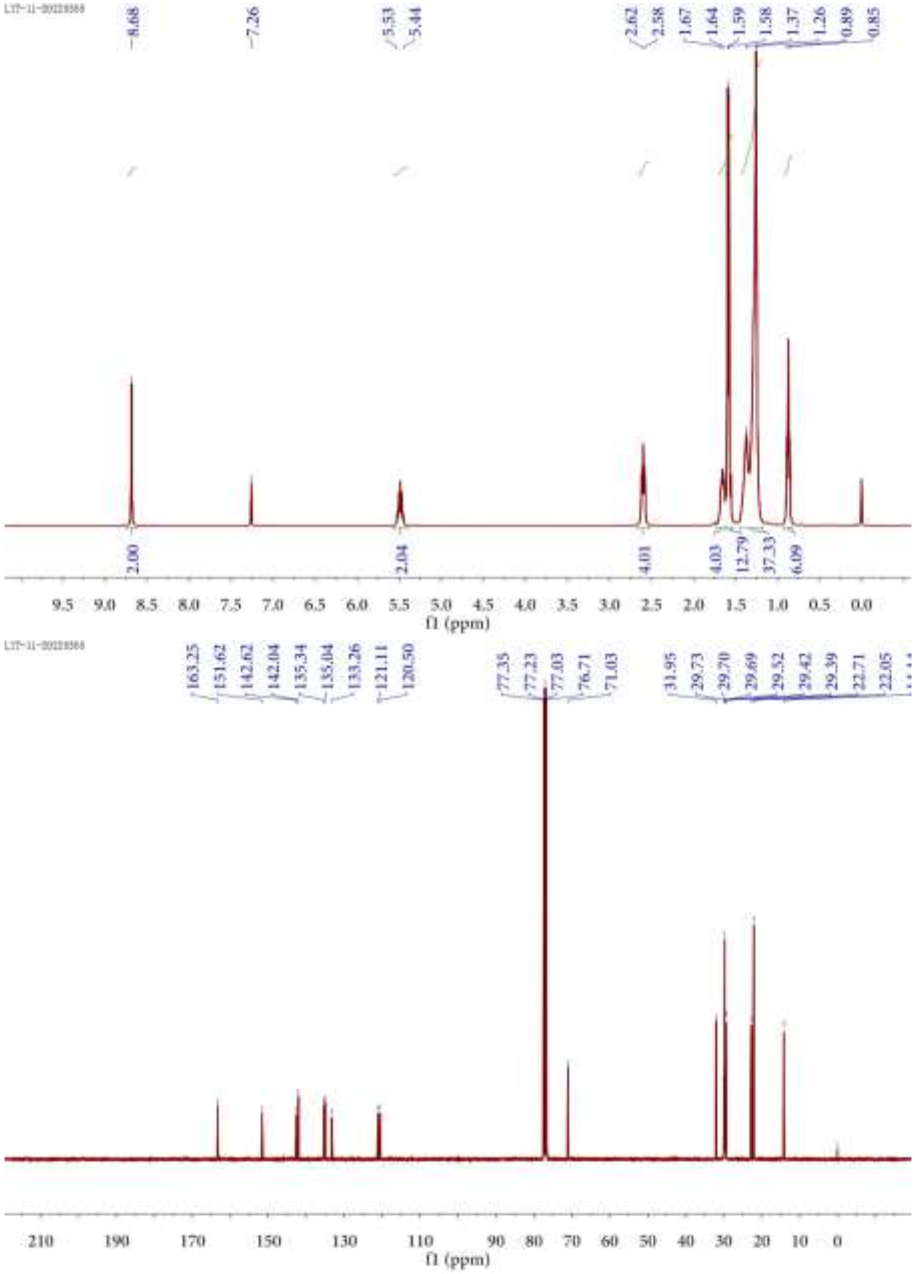

Figure S7. ^1H NMR and ^{13}C NMR spectrum of Compound 3 (400 MHz, 298 K, $CDCl_3$).

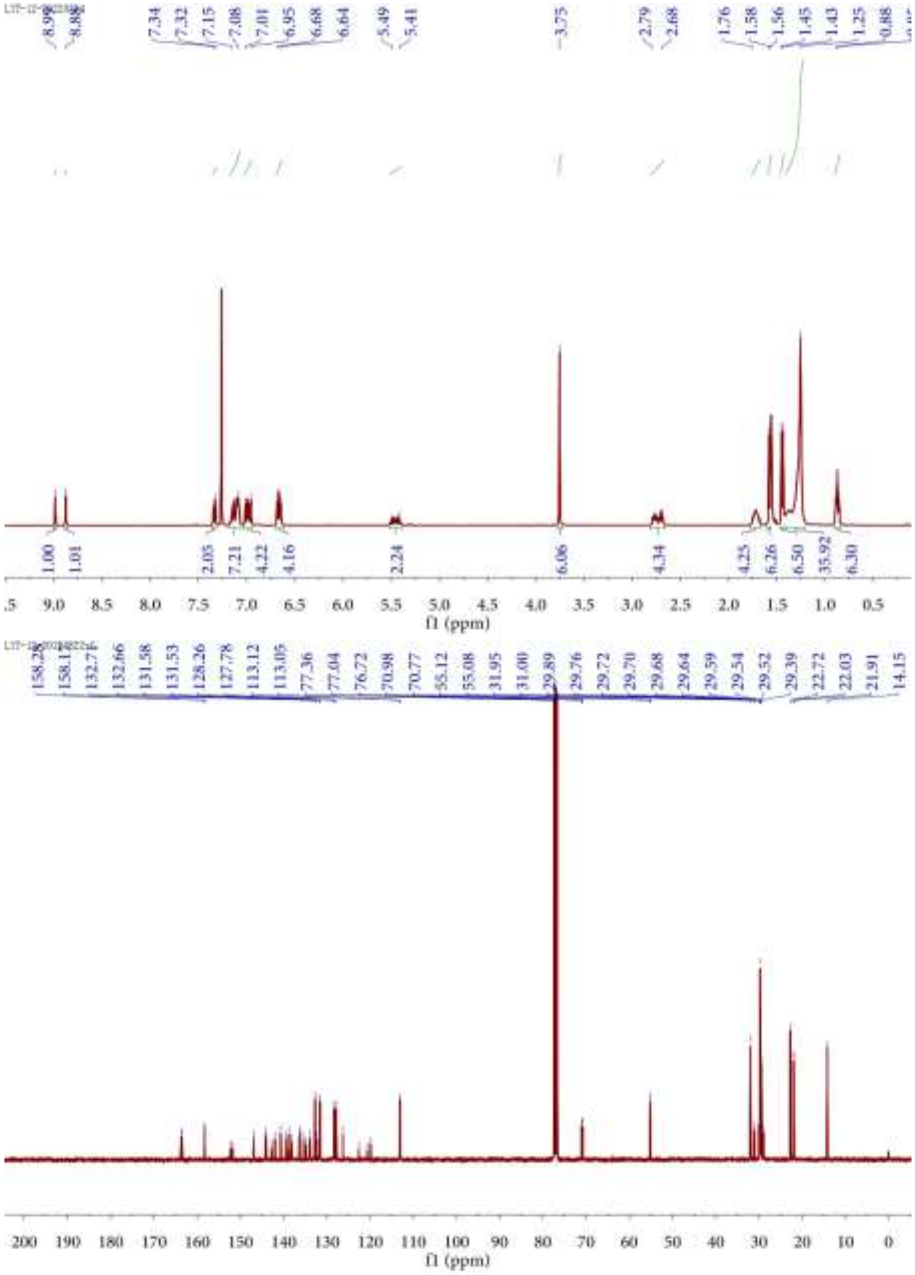

Figure S8. ^{1}H NMR and ^{13}C NMR spectrum of Compound 4 (400 MHz, 298 K, $CDCl_3$).

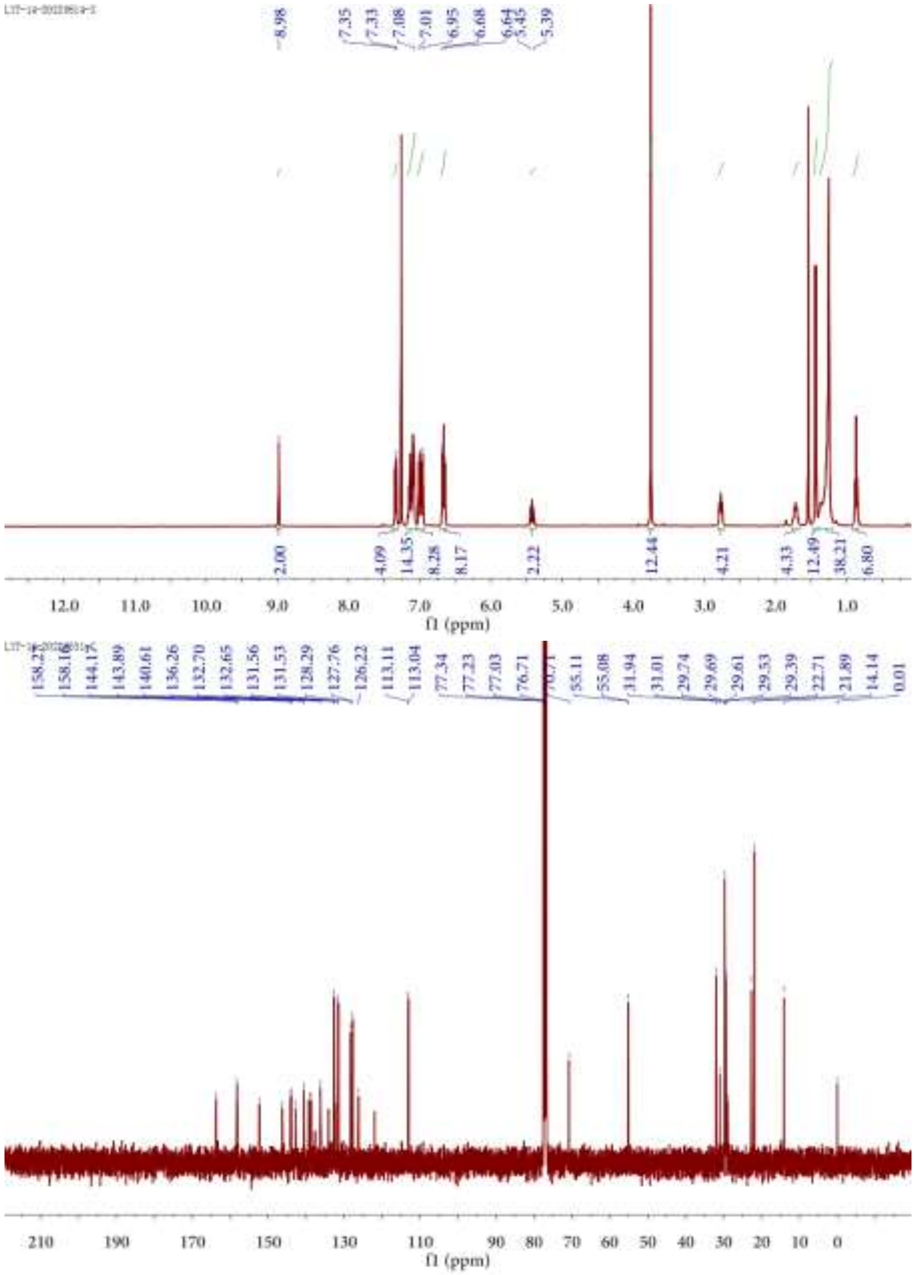

Figure S9. ^{1}H NMR and ^{13}C NMR spectrum of PTA (400 MHz, 298 K, $CDCl_3$).

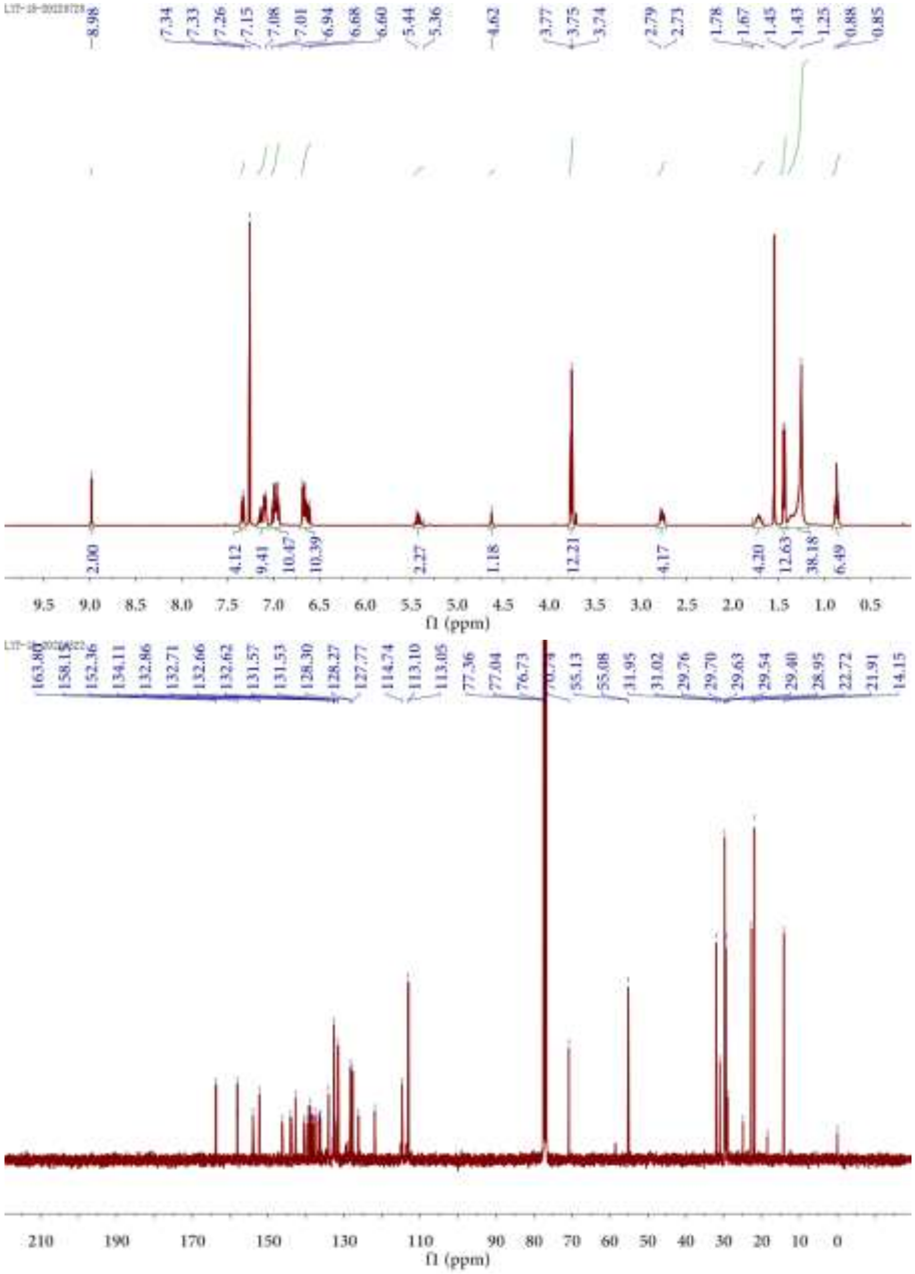

Figure S10. ^{1}H NMR and ^{13}C NMR spectrum of Compound 5 (400 MHz, 298 K, $CDCl_3$).

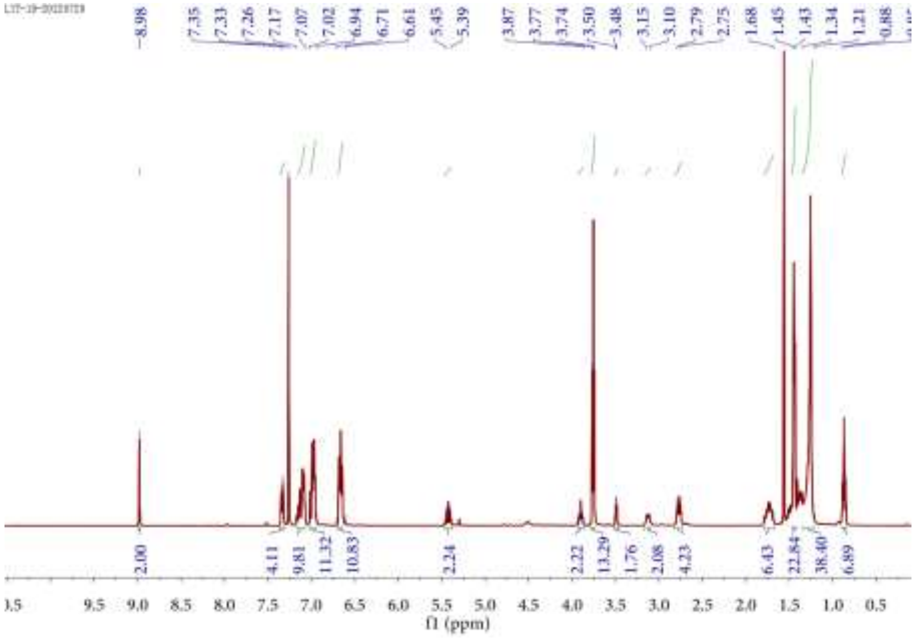

Figure S11. ^{1}H NMR spectrum of PTA-Boc (400 MHz, 298 K, $CDCl_3$).

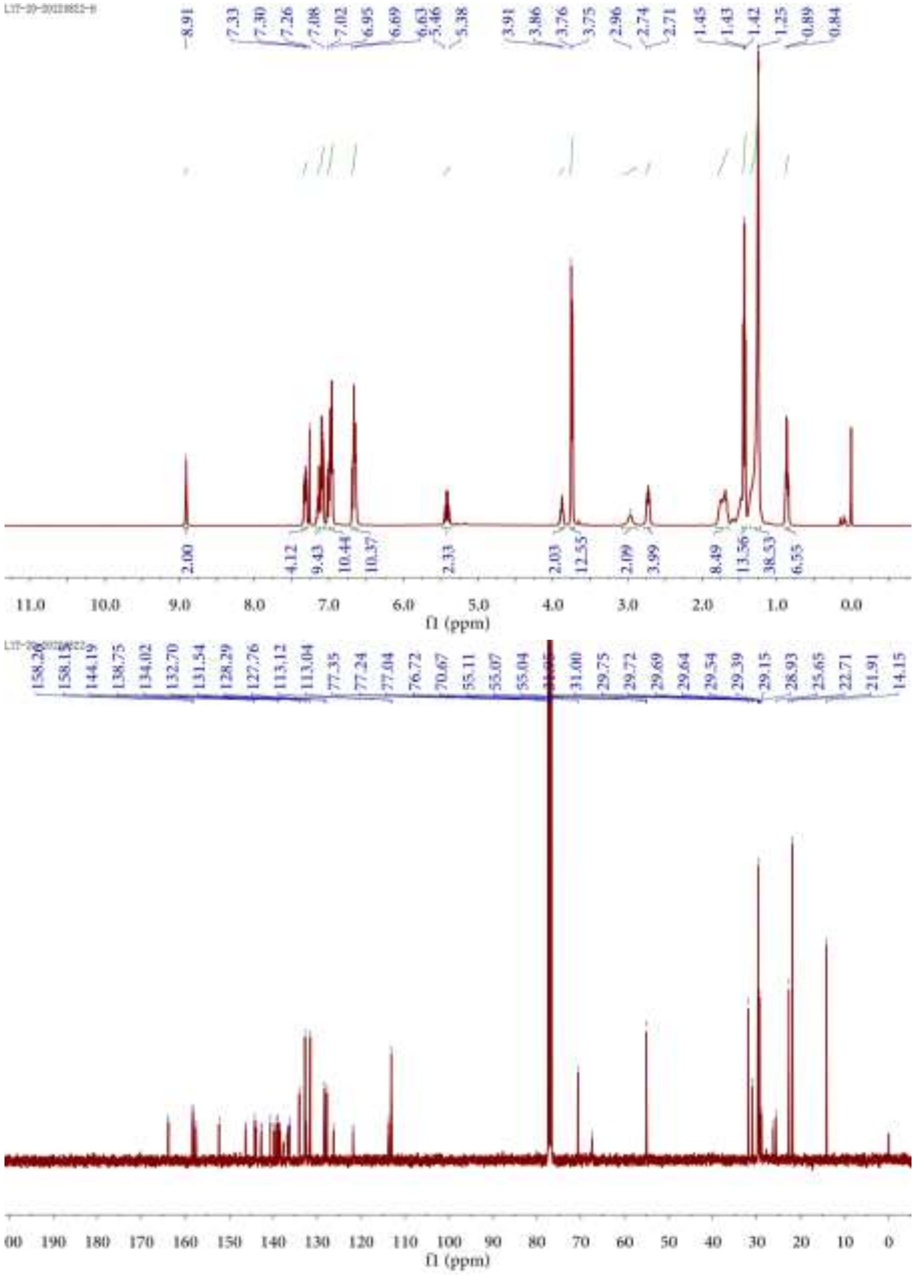

Figure S12. ^{1}H NMR and ^{13}C NMR spectrum of PTA-NH_2 (400 MHz, 298 K, $CDCl_3$).

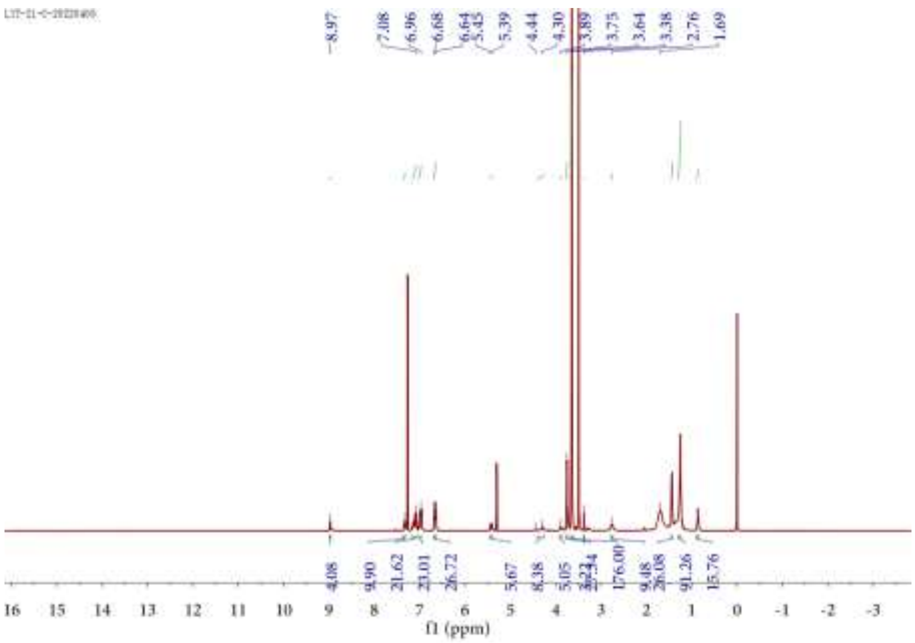

Figure S13. ^{1}H NMR spectrum of PEG_{44}-PTA_2 (400 MHz, 298 K, $CDCl_3$).

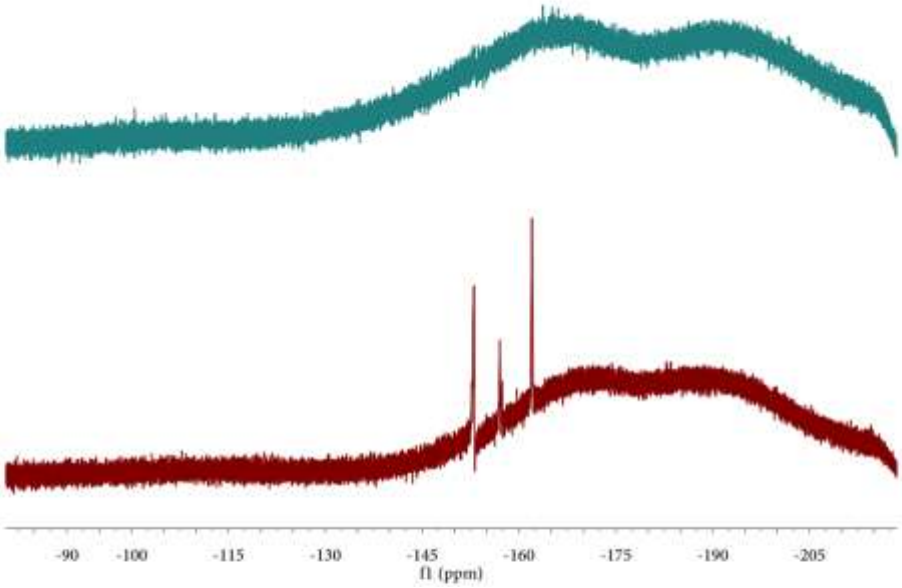

Figure S14. Representative ^{19}F NMR spectrum of PEG_{44}-PTA_2 (top) and PEG_{44}-$PTMCP_2$ (down), indicating full conversion of the TMCP group after modification.

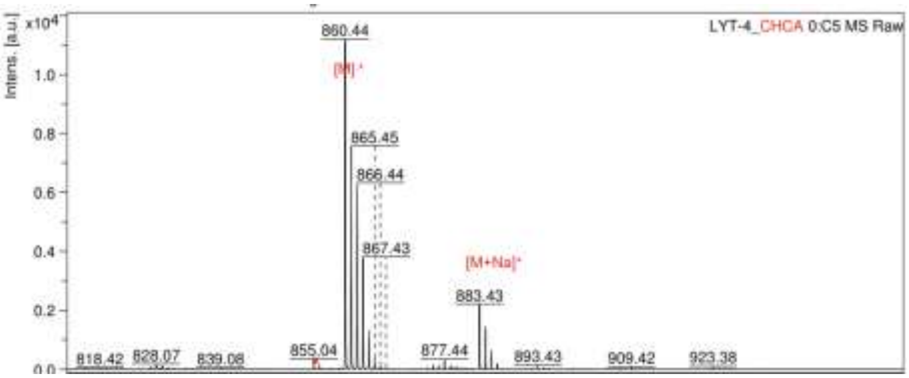

Figure S15. MALDI-TOF-MS spectrum of Compound 2.

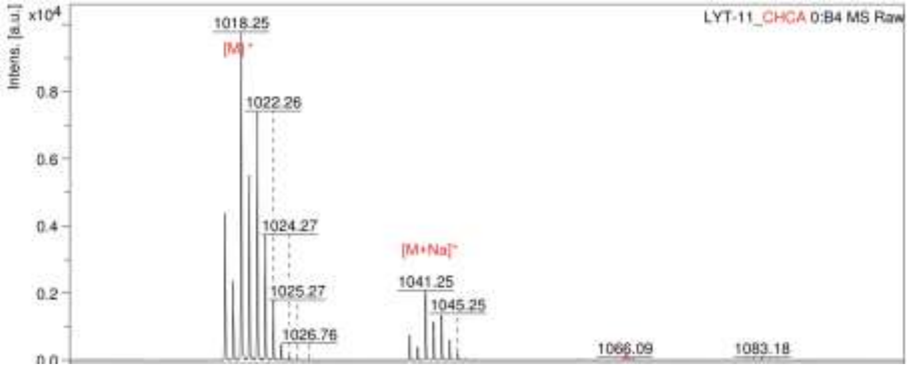

Figure S16. MALDI-TOF-MS spectrum of Compound 3.

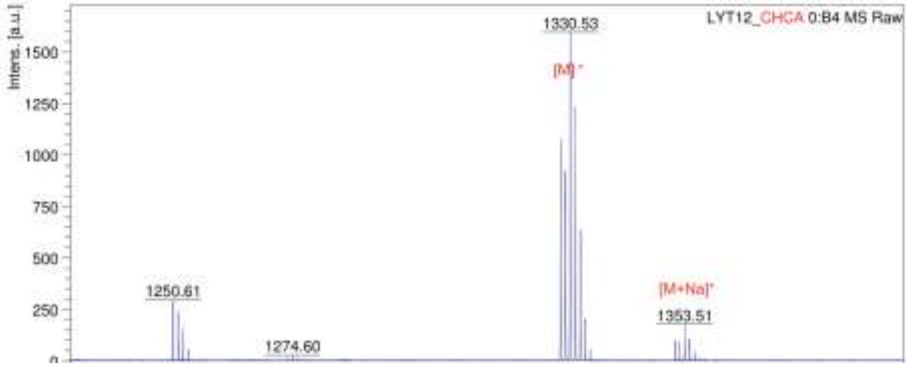

Figure S17. MALDI-TOF-MS spectrum of Compound 4.

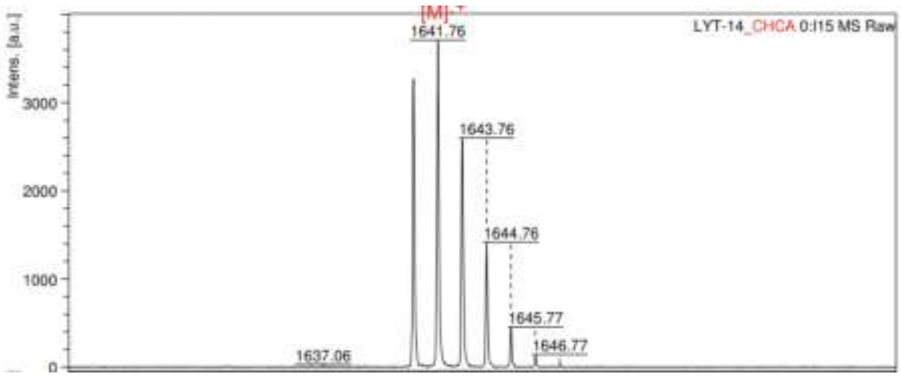

Figure S18. MALDI-TOF-MS spectrum of PTA.

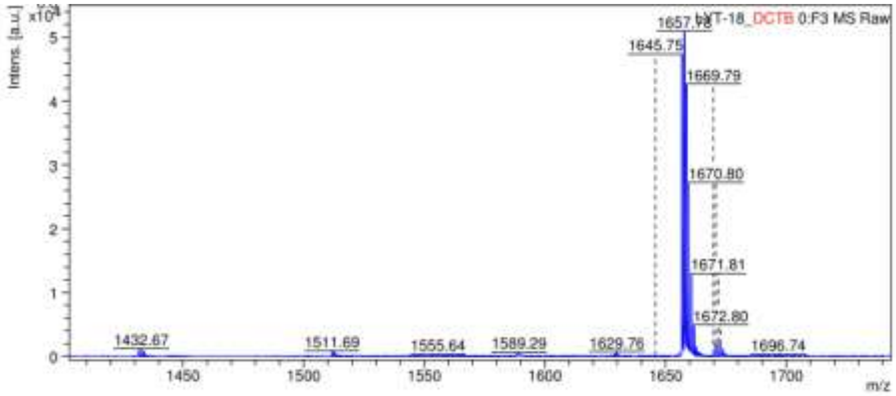

Figure S19. MALDI-TOF-MS spectrum of Compound 5.

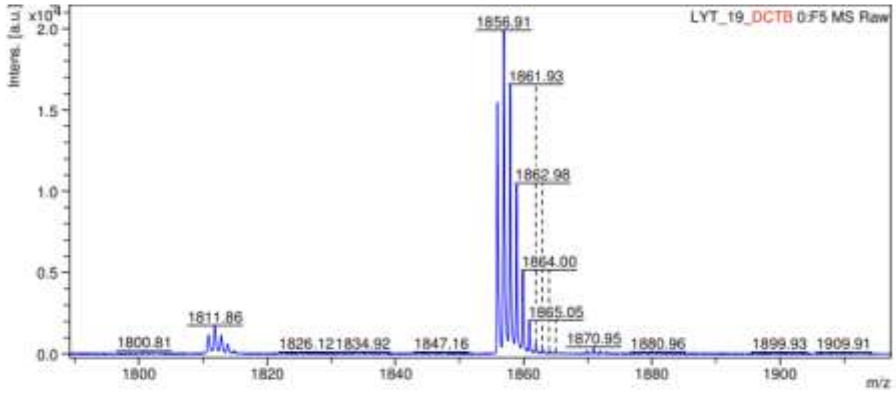

Figure S20. MALDI-TOF-MS spectrum of PTA-Boc.

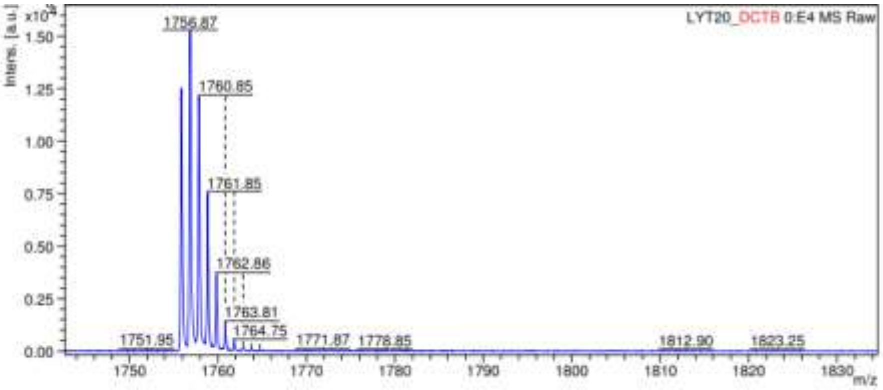

Figure S21. MALDI-TOF-MS spectrum of PTA-NH_2.

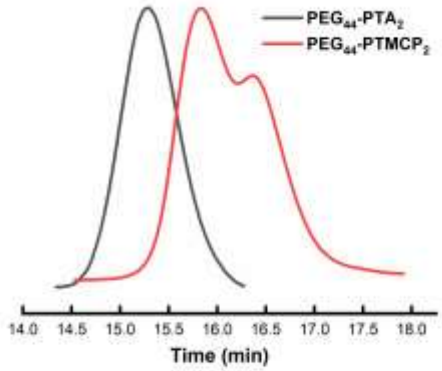

Figure S22. GPC trace of PEG_{44}-$PTMCP_2$ and PEG_{44}-PTA_2.

Table S1. GPC analysis of block copolymers.

Composition	Mn / kDa	Mw / kDa	PDI (Đ)
PEG_{44}-$PTMCP_2$	2398	2899	1.21
PEG_{44}-PTA_2	5725	6246	1.09

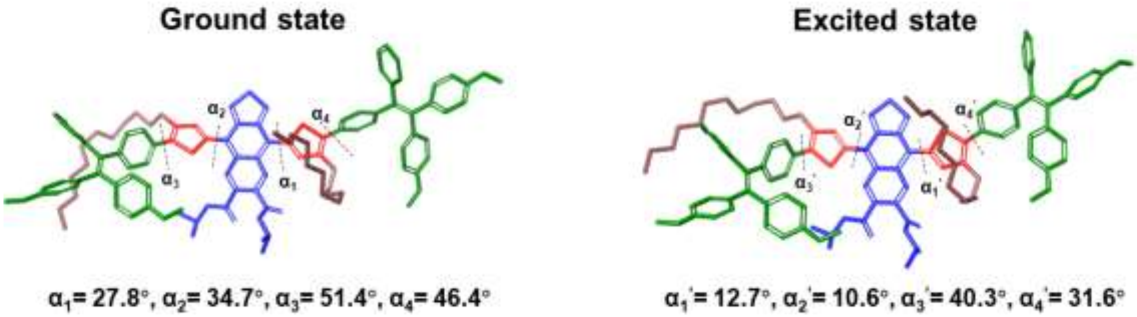

Figure S23. The DFT-optimized ground state (S_0) and excited state (S_1) geometries of PTA.

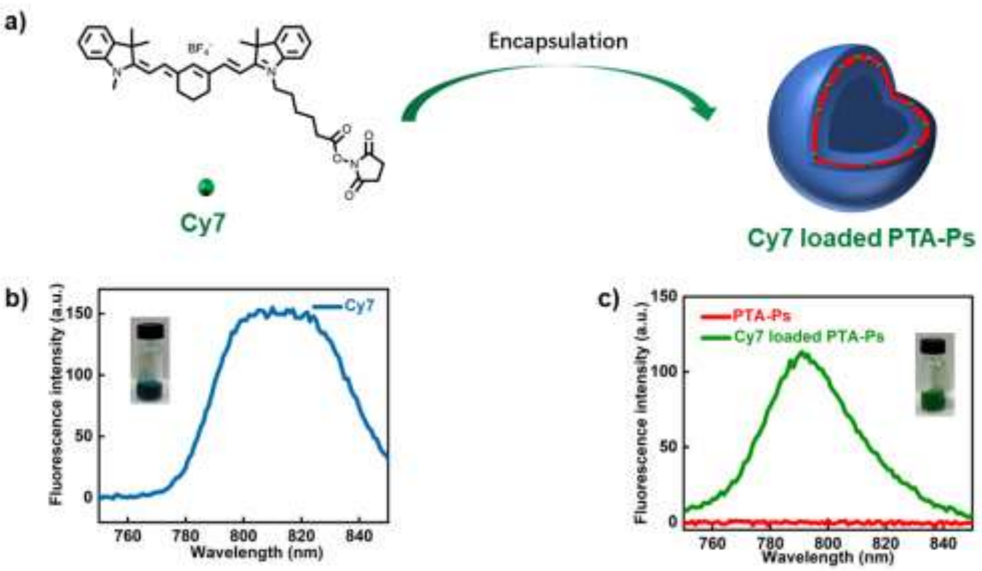

Figure S24. Characterization of Cy7 loaded PTA-Ps. a) Schematic illustration of Cy7 loaded in polymer vesicles. b) The emission spectra of pure Cy7 in THF (Ex = 720 nm). Insert is the corresponding optical image. c) The emission spectra of PTA-Ps and Cy7 loaded PTA-Ps (Ex = 720 nm). Insert is the optical image of Cy7 loaded PTA-Ps.

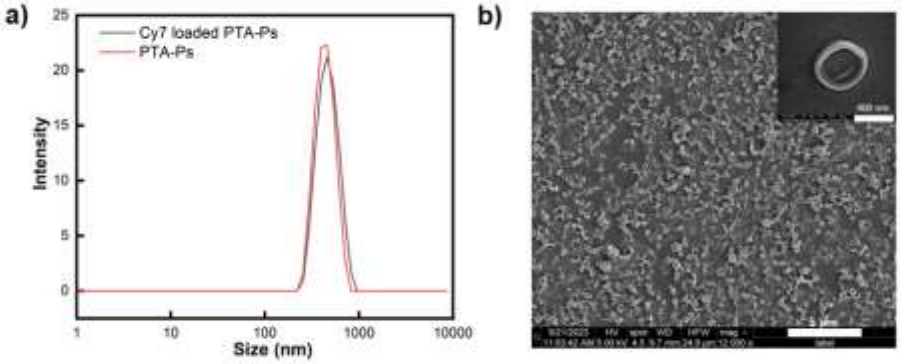

Figure. S25. a) The average size of Cy7 loaded PTA-Ps and PTA-Ps in water measured by DLS. b) SEM images of Cy7 loaded PTA-Ps. Insert is a single Cy7 loaded PTA-Ps at higher magnification. Scale bar: 5 μm / Insert: 400 nm.

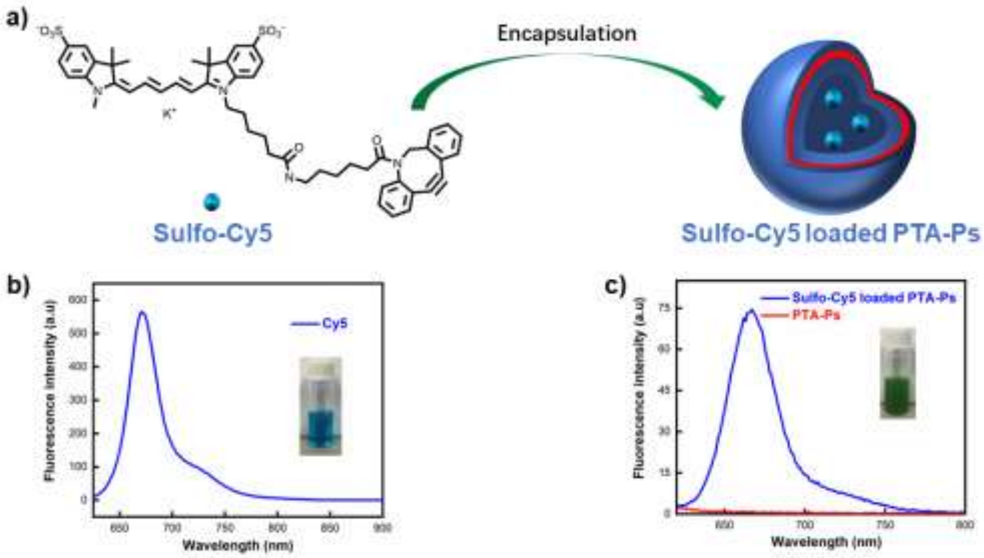

Figure S26. Characterization of Sulfo-Cy5 loaded PTA-Ps. a) Schematic illustration of Sulfo-Cy5 loaded in polymer vesicles. b) The emission spectra of pure Sulfo-Cy5 in H_2O (Ex = 600 nm). Insert is the corresponding optical image. c) The emission spectra of PTA-Ps and Sulfo-Cy5 loaded PTA-Ps (Ex = 600 nm). Insert is the corresponding optical image.

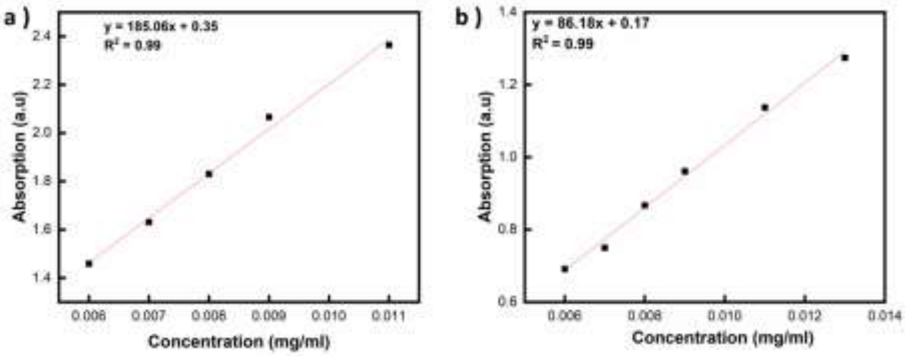

Figure S27. Absorbance-concentration calibration curve a) Sulfo-Cy5 was quantified via UV-vis-NIR spectroscopy measurement at a wavelength of 648 nm b) Cy7 was quantified via UV-vis-NIR spectroscopy measurement at a wavelength of 745 nm.

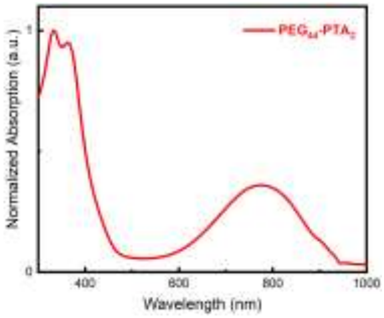

Figure S28. The normalized UV–vis–NIR absorption spectrum of PEG_{44}-PTA_2 in THF.

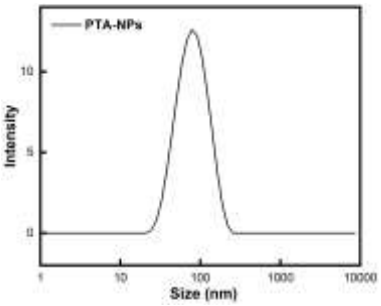

Figure S29. The average size of PTA-NPs measured by DLS.

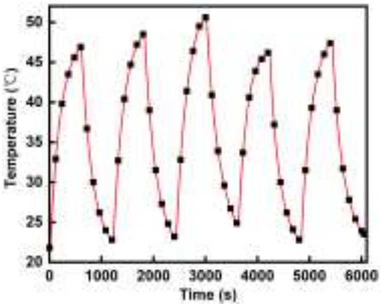

Figure S30. Photothermal stability of PTA-NPs, five circles of heating–cooling (808 nm, 0.90 W/cm^2).

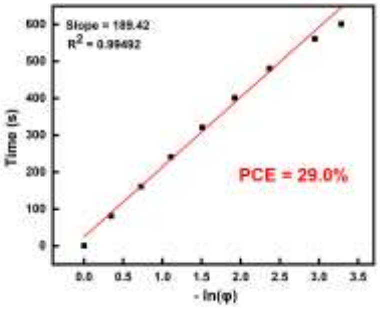

Figure S31. The time constant of PTA-NPs heat transfer from the system, calculated with the linear time data from the system cooling period versus the negative natural logarithm of the system driving force temperature. The PCE is 29.0%.

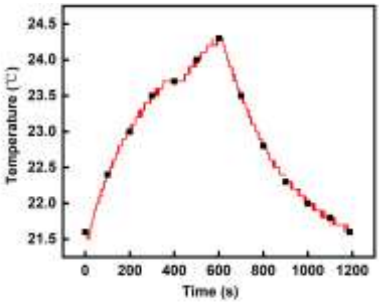

Figure S32. Plots of temperature vs time of pure water during heating via laser irradiation (808 nm, 0.90 W/cm^2) and cooling (laser off).

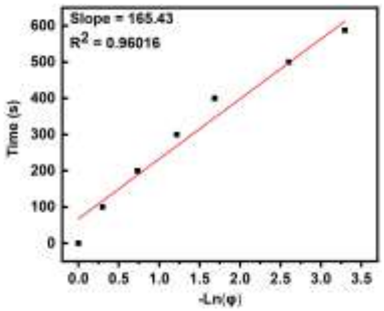

Figure S33. The time constant of water heat transfer from the system, calculated with the linear time data from the cooling period.

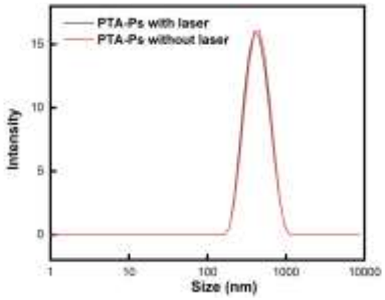

Figure S34. The average size of PTA-Ps (with and without 808 nm laser irradiation for 10 min) measured by DLS.

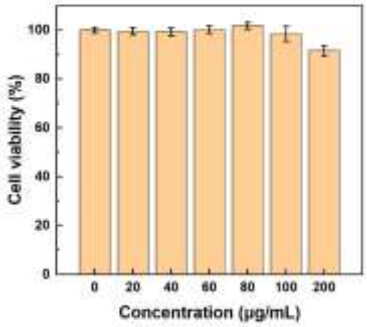

Figure S35. Cytotoxicity test of PTA-Ps upon incubation with HeLa cells for 24 h.

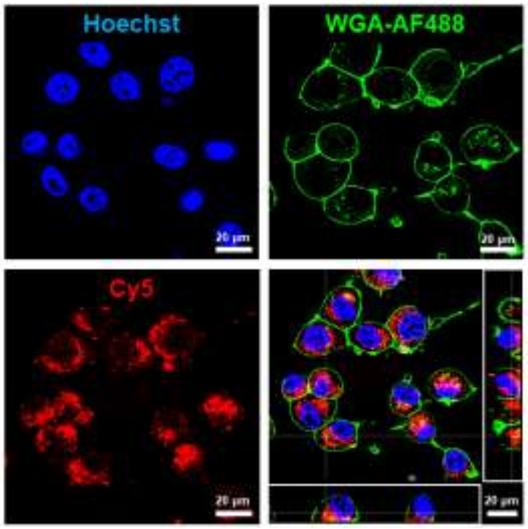

Figure S36. CLSM images of HeLa cells after incubation with Cy5 loaded PTA-Ps (200 ug/ml) for 19 h, followed by staining the cell membrane and nucleus with WGA-AF488 and Hoechst 33342. Scale bar: 20 μm

5. References

1. Qi, J.; Duan, X.; Cai, Y.; Jia, S.; Chen, C.; Zhao, Z.; Li, Y.; Peng, H. Q.; Kwok, R. T. K.; Lam, J. W. Y.; Ding, D.; Tang, B. Z. Simultaneously boosting the conjugation, brightness and solubility of organic fluorophores by using AIEgens. *Chem. Sci*. 2020, *11*, 8438-8447
2. Luo, Y.; Lan, Y.; Peng, L.; Qiu, T.; Li, Q.; Hou, Q.; Liu, C.; Zhang, J.; Cai, Y. P.; Liu, S. Acrylate-Substituted Thiadiazoloquinoxaline Yields Ultralow Band Gap (0.56 eV) Conjugated Polymers for Efficient Photoacoustic Imaging. *ACS Appl. Polym. Mater*. 2021, *3*, 3247-3253.
3. Cao, S.; Shao, J.; Wu, H.; Song, S.; De Martino, M. T.; Pijpers, I. A. B.; Friedrich, H.; Abdelmohsen, L. K. E. A.; Williams, D. S.; Van Hest, J. C. M. Photoactivated nanomotors via aggregation induced emission for enhanced phototherapy. *Nat. Commun*. 2021, *12*, 1-10.
4. Liu, S.; Zhou, X.; Zhang, H.; Ou, H.; Lam, J. Y.; Liu, Y.; Shi, L.; Ding, D.; Tang, B. Z. Molecular Motion in Aggregates: Manipulating TICT for Boosting Photothermal Theranostics. *J. Am. Chem. Soc*. 2019, *141*, 5359-5368.
5. D'Angelo, N. A.; Câmara, M. C. C.; Noronha, M. A.; Grotto, D.; Chorilli, M.; Lourenço, F. R.; Rangel-Yagui, C.; Lopes, A. M. Development of PEG-PCL-based polymersomes through design of experiments for co-encapsulation of vemurafenib and doxorubicin as chemotherapeutic drugs. *J. Mol. Liq*. 2022, *349*, 118166.
6. Xi, D.; Xiao. M.; Cao, J.; Zhao, L.; Xu, N.; Long, S.; Fan, J.; Shao, K.; Sun, W.; Yan, X.; Peng, X. NIR light-driving barrier-free group rotation in nanoparticles with an 88.3% photothermal conversion efficiency for photothermal therapy. *Adv. Mater*. 2020, *32*,1907855.
7. User Manual Schrödinger. Inc. New York NY 2019.
8. Roos, K.; Wu, C.; Damm, W.; Reboul, M.; Stevenson, J. M.; Lu, C.; Dahlgren, M. K.; Mondal, S.; Chen, W.; Wang, L.; Abel, R.; Friesner, R. A.; Harder, E. D. OPLS3e: Extending force field coverage for drug-like small molecules. *J. Chem. Theory Comput*. 2019, *15*, 1863-1874.
9. Marten, B.; Kim, K.; Cortis, C.; Friesner, R. A.; Murphy, R. B.; Ringnalda, M. N.; Sitkoff, D.; Honig, B. New model for calculation of solvation free energies: correction of self-consistent reaction field continuum dielectric theory for short-range hydrogen-bonding effects. *J. Phys. Chem*. 1996, *100*, 11775-11788.
10. Mohamadi, F.; Richards, N. G. J.; Guida, W. C.; Liskamp, R.; Lipton, M.; Caufield, C.; Chang, G.; Hendrickson, T.; Still, W. C. MacroModel-An Integrated Software System for Modeling Organic and Bioorganic Molecules Using Molecular Mechanics. *J. Comput. Chem*. 1990, *11*, 440–467.
11. Bachrach, S. M.; Jaguar, 5.5. Schrödinger. *J. Am. Chem. Soc*. 2004, *126*, 5018.
12. Lee, C.; Yang, W.; Parr, R. G. Development of the Colle-Salvetti correlation-energy formula into a functional of the electron density. *Phys. Rev. B*, 1988, *37*, 785.
13. Becke, A. D. New Mixing of Hartree-Fock and Local Density-Functional Theories. *J. Chem. Phys*. 1993, *98*, 1372–1377.

14. Hay, P. J.; Wadt, W. R. Ab Initio Effective Core Potentials for Molecular Calculations. Potentials for K to Au Including the Outermost Core Orbitals. *J. Chem. Phys*. 1985, *82*, 299–310.
